# Associations of *MTHFR* Gene Polymorphisms with Hypertension and Hypertension in Pregnancy: A Meta-Analysis from 114 Studies with 15411 Cases and 21970 Controls

**DOI:** 10.1371/journal.pone.0087497

**Published:** 2014-02-05

**Authors:** Boyi Yang, Shujun Fan, Xueyuan Zhi, Yongfang Li, Yuyan Liu, Da Wang, Miao He, Yongyong Hou, Quanmei Zheng, Guifan Sun

**Affiliations:** 1 Environment and Non-Communicable Diseases Research Center, School of Public Health, China Medical University, Shenyang, China; 2 Department of Medical and Molecular Genetics, School of Medicine, Indiana University, Indianapolis, Indiana, United States of America; Tabriz University of Medical Sciences, Iran (islamic Republic Of)

## Abstract

**Background:**

Several epidemiological studies have investigated the associations of methylenetetrahydrofolate reductase (*MTHFR*) C677T and A1298C polymorphisms with hypertension (H) or hypertension in pregnancy (HIP). However, the results were controversial. We therefore performed a comprehensive meta-analysis to provide empirical evidences on the associations.

**Methodologies:**

The English and Chinese databases were systematically searched to identify relevant studies. Odds ratios (ORs) and 95% confidence intervals (CIs) were calculated to evaluate the strength of the associations. Meta-regression, subgroup analysis, sensitivity analysis, cumulative meta-analysis and assessment of publication bias were performed in our study.

**Principal Findings:**

A total of 114 studies with 15411 cases and 21970 controls were included, 111 studies with 15094 cases and 21633 controls for the C677T polymorphism and 21 with 2533 cases and 2976 controls for the A1298C polymorphism. Overall, the C677T polymorphism was significantly associated with H and HIP (H & HIP: OR = 1.26, 95% CI = 1.17–1.34; H: OR = 1.36, 95% CI = 1.20–1.53; HIP: OR = 1.21, 95% CI = 1.08–1.32). Stratified analysis by ethnicity revealed a significant association among East Asians and Caucasians, but not among Latinos, Black Africans, and Indians and Sri Lankans. In the stratified analyses according to source of controls, genotyping method, sample size and study quality, significant associations were observed in all the subgroups, with the exception of population based subgroup in H studies and large sample size and “others” genotyping method subgroups in HIP studies. For the A1298C polymorphism, no significant association was observed either in overall or subgroup analysis under all genetic models.

**Conclusions:**

This meta-analysis suggests that the *MTHFR* C677T rather than A1298C polymorphism may be associated with H & HIP, especially among East Asians and Caucasians.

## Introduction

Hypertension (H), whose prevalence has dramatically increased in recent years, is a major risk factor for many disorders including stroke, cardiovascular diseases and renal failure and ultimately increases mortality worldwide [1]. The development of H is influenced by genetic, environmental, demographic factors and their interactions [2]. Current evidences suggest that 30–50% of variation of blood pressure levels could be attributed to genetic factors [3]. Therefore, identification of H susceptibility genes will help clarify the pathogenesis of the disease and provide new therapeutic and preventive strategies [3]. In the last decade, exhaustive efforts have been devoted to unraveling the genetic underpinning of H, and hundreds of genes and polymorphisms have been hypothesized to be involved in the pathogenesis of the disease [4]–[6]. Among them, C677T and A1298C polymorphisms in methylenetetrahydrofolate reductase (*MTHFR*) gene have been assessed as potential candidates.

*MTHFR* is an enzyme that catalyzes the reduction of 5,10-methylenetetrahydrofolate to 5-methytetrahydrofolate, the carbon donor for the remethylation of homocysteine (Hcy) to methionine [7]. The *MTHFR* gene is localized on chromosome 1 at 1p36.6 [8]. The C677T polymorphism is a C to T transition at base pair 677 resulting an alanine to valine substitution, and the A1298C polymorphism is an A to C transition at base pair 1298 leading to a glutamate to alanine substitution. The prevalence of the two polymorphisms varies in different geographical regions and ethnic groups [9], [10]. The variant genotypes of them have been confirmed to reduce enzyme activity and decrease folate levels, and subsequently result in hyperhomocysteinemia (HHcy) [11], [12]. HHcy has been linked to H and hypertension in pregnancy (HIP) because it may induce arteriolar constriction, renal dysfunction and increased sodium reabsorption, and also increase arterial stiffness and oxidative stress [13]–[15]. Therefore, the *MTHFR* C677T and A1298C polymorphisms as common genetic causes for HHcy are expected to be associated with hypertension and hypertension in pregnancy (H & HIP).

Numerous epidemiological studies were conducted in recent years to evaluate the associations between the *MTHFR* C677T and A1298C polymorphisms and H & HIP. However, the results were conflicting or inconclusive, presumably due to small sample size in each published study, various genetic backgrounds and possible selection bias. Meta-analysis is a widely used statistical method in medical research, especially for a topic being extensively studied while controversial results are being reported. Two meta-analyses, one by Qian et al. [16], the other by Niu et al. [17], were performed in 2007 and 2011, respectively, to investigate the associations of the C677T polymorphism with H & HIP and significant results were reported. However, Niu et al.’s [17] meta-analysis only included studies in the analysis of Chinese population. Additionally, new epidemiological studies have recently been conducted to estimate the associations of the *MTHFR* C677T and A1298C polymorphisms with H and/or HIP in different populations and provide new evidences that were not included in these previous meta-analyses. Moreover, both meta-analyses did not address the associations of the A1298C polymorphism with H and/or HIP. To provide a more comprehensive assessment of the associations of the *MTHFR* C677T and A1298C polymorphisms with H & HIP in worldwide populations, we carried out a meta-analysis of all eligible studies.

## Materials and Methods

### Search Strategy and Inclusion Criteria

All studies reporting the relationships of the *MTHFR* C677T and A1298C polymorphisms with H or HIP published before December 10, 2013 were identified by computerized searches in databases including Pubmed, Embase, ISI Web of Science, China Biological Medicine Database (CBM), Wanfang, China National Knowledge Infrastructure (CNKI), and Chongqing VIP Chinese Science and Technology Periodical Database (VIP). The search strategies were based on combinations of the following key words: (“methylenetetrahydrofolate reductase” or “*MTHFR*”) and (“hypertension” or “hypertension in pregnancy” or “pregnancy induced hypertension” or “preeclampsia” or “eclampsia” or “gestational hypertension”) and (“gene” or “allele” or “genotype” or “mutation” or “variant” or “variation” or “polymorphism”). The reference lists of retrieved articles were also hand searched for additional articles.

Qualified studies had to meet the following criteria: (1) evaluation of the *MTHFR* C677T and/or A1298C polymorphisms and H or HIP; (2) hypertensive patients were diagnosed according to the criteria of SBP≥140 mmHg or DBP≥90 mmHg and the controls were healthy individuals; (3) case-control or cohort study, regardless of sample size, using a hospital based or a population based design; (4) sufficient published data for estimating the Odds Ratio (OR) and 95% confidence interval (CI); (5) for duplicate publication, the most recent or largest study was selected.

### Data Extraction

Two reviewers (Boyi Yang and Shujun Fan) independently extracted the following information from each included study: the first author’s name, publication year, sample size, source of controls, ethnicity, genotyping method, matching variables of controls with cases, H type (H vs. HIP), age, gender proportion, and counts of alleles and genotypes in both cases and controls.

### Quality Assessment

Two authors (Boyi Yang and Xueyuan Zhi) independently assessed the quality of the included studies according to Newcastle Ottawa Scale (NOS) (www.ohri. ca/programs/clinical_epidemiology/oxford.asp). This scale consists of three parts relating to selection, comparability and ascertainment of exposure. A maximum nine scores could be given to the highest quality studies. A score of five or more was regarded as “high quality”; otherwise, the study was regarded as “low quality”.

### Statistical Analysis

All statistic tests performed in this study were two tailed and *P*<0.05 was taken as statistically significant, unless otherwise stated. Statistic analyses were performed using STATA package version 11.0 program (Stata corp, College Station, TX). Hardy-Weinberg equilibrium (HWE) in controls was calculated again in our meta-analysis. The chi-square goodness of fit was used to test deviation from HWE.

Crude ORs with corresponding 95% CIs were calculated to estimate the strength of the associations of the *MTHFR* C677T and A1298C polymorphisms with H and/or HIP. The significance of the pooled OR was determined by the Z test. Pooled frequency analysis was carried out using the method suggested by Thakkinstian [18]. The overall pooled ORs were calculated using allele contrast model, dominant model and recessive model. Moreover, comparisons of OR_1_ (AA vs. aa), OR_2_ (Aa vs. aa) and OR_3_ (AA vs. Aa) were explored with A as the risk allele. The above pairwise differences were used to determine the most appropriate genetic model. If OR_1_ = OR_3_ ≠ 1 and OR_2_ = 1, then a recessive model is selected. If OR_1_ = OR_2_ ≠ 1 and OR_3_ = 1, then a dominant model is selected. If OR_2_ = 1/OR_3_ ≠ 1 and OR_1_ = 1, then a complete overdominant model is selected. If OR_1_> OR_2_>1 and OR_1_> OR_3_>1 (or OR_1_< OR_2_<1 and OR_1_< OR_3_<1), then a codominant model is selected [19]. Additionally, if some genotypes were very rare or could not be identified in either case or control group in some studies, a recessive or dominant model is selected to combine rare homozygous and heterozygous [20].

Between-study heterogeneity was calculated by Cochran’s Chi-square based Q-test [21]. Simultaneously, it was also detected using the *I* ^2^ statistic (*I* ^2^ = 0–25% represents no heterogeneity; *I* ^2^ = 25–50% represents moderate heterogeneity; *I* ^2^ = 50–75% represents large heterogeneity; *I* ^2^ = 75–100% represents extreme heterogeneity) [22]. If the between-study heterogeneity was statistically significant (*P*<0.10 for Q-test or I^2^>50%), the Dersimonian and Laird random effects model was used; otherwise, the Mantel Haenszel method fixed effects model was applied [23]. Subgroup analysis based on ethnicity (East Asians, Caucasians, Latinos, Indians and Sri Lankans, Black Africans), source of controls (population based vs. hospital based), genotyping method (polymerase chain reaction-restriction fragment length polymorphism (PCR-RFLP) vs. “others”), sample size (studies with ≥ median number of participants vs. studies with < median number) and study quality (high quality vs. low quality), respectively, were also performed under the most appropriate genetic model. Furthermore, meta-regression was employed to explore potential sources of heterogeneity including publication date, ethnicity, genotyping method, source of controls, study quality and sample size [24]. To explore the dynamic trends as studies accumulated over time, cumulative meta-analysis was performed by date of publication [25]. Sensitivity analysis was also conducted to examine the influence of excluding each study or some specific studies on the overall estimate [25]. Finally, potential publication bias was assessed using funnel plot and Egger’s regression test [26].

## Results

### Study Characteristics

**Figure 1:**
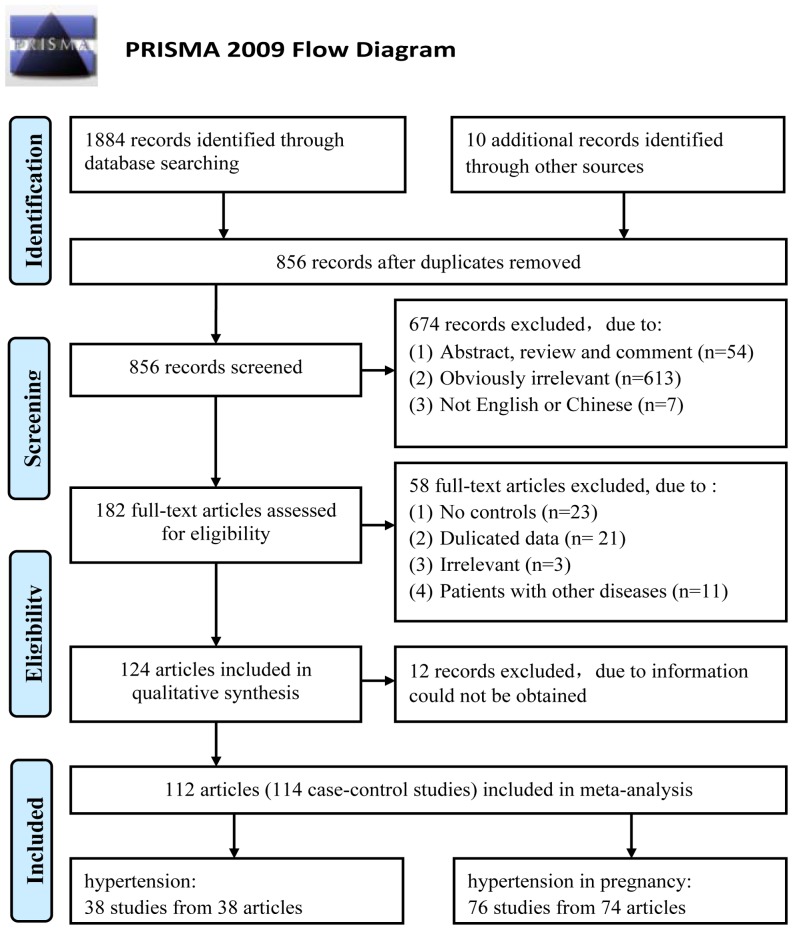
Flow diagram of study selection process in this meta-analysis.

The combined search yielded 1884 articles. After the removal of overlapping articles and those did not meet our inclusion criteria, a total of 112 articles [27]–[138] including 114 studies with 15411 cases and 21970 controls were finally included in the meta-analysis (Figure 1). One hunderd and eleven studies dealt with C677T. The sample sizes ranged from 39 to 2104 with a median of 225. Twenty one studies dealt with A1298C. The sample sizes ranged from 58 to 754 with a median of 170. The main characteristics of the included studies are presented in Table S1. Among all studies, 42 studies were performed among East Asians [27], [28], [33], [37], [42], [43], [48], [50], [54], [56], [57], [60], [65], [73], [75], [78], [79], [81], [86]–[90], [94]–[97], [102], [103], [105]–[107], [115]–[118], [122], [124], [131], [132], [134], [136], 54 among Caucasians [29], [31], [32], [34], [35], [38]–[41], [44]–[47], [49], [51]–[53], [55], [59], [63], [64], [66], [67], [69]–[72], [74], [76], [77], [80], [82], [84], [92], [93], [98]–[101], [109], [110], [112]–[114], [119]–[121], [125], [126], [130], [133], [135], [137], 10 among Latinos [58], [61], [68], [83], [91], [104], [108], [127], [128], [138], five among Indians and Sri Lankans [53], [85], [111], [123], [129] and three among Black Africans [30], [36], [62]. Thirty eight studies focused on H [28], [37], [45], [54], [55], [64]–[66], [75], [78], [80], [81], [85], [87], [88], [90], [92], [95], [98], [99], [102], [105], [106], [114]–[118], [134], [119], [120], [122], [124], [128], [131], [132], [136], [137] and 76 studies focused on HIP [27], [29]–[36], [38], [39]–[44], [46]–[53], [56]–[63], [67]–[74], [76], [77], [79], [82]–[84], [86], [89], [91], [93], [94], [96], [97], [100], [101], [103], [104], [107]–[113], [121], [123], [125]–[127], [129], [130], [133], [135], [138]. The sources of controls were hospital based in 91 studies and were population based in 23 studies. PCR-RFLP was the most commonly used genotyping method in these included studies. Genotype and allele frequencies, HWE and NOS scale information are presented in Table S2 and Table S3. Of the total 114 studies, 20 different studies [27], [35], [40], [45], [73], [79], [85], [87], [94], [96], [105], [107], [109], [110], [115], [116], [122], [129], [135] showed significant deviations from HWE (18 studies concerned C677T and two studies concerned A1298C). Thirteen studies only reported combined genotypes (CC+CT, CT+TT, AC+CC), thus HWE could not be evaluated (12 studies concerned C677T [29], [41], [49], [51], [67], [69], [71], [77], [100], [101], [114], [119] and one study concerned A1298C [119]). According to NOS scale, there were 100 studies with high quality and 14 with low quality.

### Frequency of Risk Allele in the Control Population

**Figure 2:**
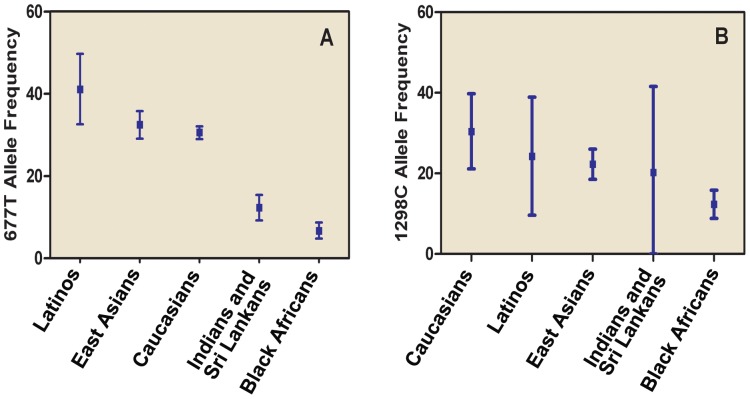
Pooled frequencies of the *MTHFR* 677T allele and 1298C allele among controls stratified by ethnicity.

Figure 2 shows the pooled frequencies of the 677T and 1298C alleles in the control populations stratified by ethnicity. The frequencies of the 677T allele varied among ethnicities: the pooled 677T allele frequency was highest among Latinos (41.5%, 95% CI = 34.0–49.0%), followed by East Asians (33.0%, 95% CI = 29.7–36.3%), Caucasians (30.1%, 95% CI = 28.5–31.6%), Indians and Sri Lankans (12.3%, 95% CI = 9.2–15.4%) and Black Africans (6.7%, 95% CI = 4.8–8.7%). The pooled 1298C allele frequencies also showed heterogeneity among different ethnicities: high among Caucasians (30.4%, 95% CI = 21.1–39.8%), intermediate among Latinos (24.2%, 95% CI = 9.6–38.9%), East Asians (22.3%, 95% CI = 18.5–26.0%) and Indians and Sri Lankans (20.2%, 95%CI = 0–41.6%), and low among Black Africans (12.3%, 95% CI = 8.8–15.8%).

### Quantitative Synthesis and Heterogeneity Analysis

#### Association of *MTHFR* C677T polymorphism with H & HIP

We firstly pooled all the studies (111 studies with 15094 cases and 21633 controls) involving both H and HIP to estimate the associations between the diseases and the *MTHFR* C677T polymorphism. Table 1 summarizes the ORs with corresponding 95% CIs for the relationships of the polymorphism with H & HIP in homozygous codominant, heterozygous codominant, dominant, recessive and allele contrast genetic models (Figure S1–S5). The dominant model was determined according to the principle of genetic model selection [19], [20]. The summary results indicated a significant association between the *MTHFR* C677T polymorphism and H & HIP. For the dominant model, the pooled OR using random effects model was 1.26 (95% CI = 1.17–1.34) (Table 1 and Figure S3). Subgroup analysis for ethnicity indicated that the polymorphism was associated with H & HIP among East Asians and Caucasians, but not among Latinos, Black Africans, and Indians and Sri Lankans. Additionally, when stratified analyses were conducted according to source of controls, genotyping method, sample size and study quality, the polymorphism was significantly associated with H & HIP in all the subgroups (Table 2). Significant heterogeneity was observed, thus a meta-regression was performed subsequently to explore the heterogeneity sources. The results of meta-regression indicated that ethnicity had a statistical significance (*P = *0.043), while the H type (*P* = 0.829), year of publication (*P* = 0.293), source of controls (*P* = 0.400), genotyping method (*P* = 0.439) and sample size (*P* = 0.579) had no statistical significance.

**Table 1:** Summarized ORs with 95% CIs for the associations of *MTHFR* polymorphisms with H and HIP.

Polymorphism	Genetic model		n	Statistical model	OR (95% CI)	*P*_z_	I^2^ (%)	*P*_h_	*P*_e_
C677T									
	Allele contrast	H & HIP	99	Random	1.23 (1.16–1.31)	<0.001	56.0	<0.001	0.280
		H	34	Random	1.30 (1.18–1.43)	<0.001	64.1	<0.001	0.816
		HIP	65	Random	1.19 (1.10–1.29)	<0.001	48.7	<0.001	0.149
	Homozygous	H& HIP	99	Random	1.47 (1.30–1.66)	<0.001	41.5	<0.001	0.362
	codominant	H	34	Random	1.63 (1.34–1.98)	<0.001	54.1	<0.001	0.497
		HIP	65	Random	1.37(1.18–1.58)	<0.001	31.0	0.011	0.495
	Heterozygous	H & HIP	99	Random	1.18 (1.10–1.27)	<0.001	38.4	<0.001	0.059
	codominant	H	34	Random	1.25 (1.11–1.40)	<0.001	43.1	0.005	0.979
		HIP	65	Random	1.14 (1.03–1.26)	0.009	34.3	0.004	0.052
	Dominant	H & HIP	101	Random	1.26 (1.17–1.34)	<0.001	48.2	<0.001	0.711
		H	35	Random	1.36 (1.20–1.53)	<0.001	55.0	<0.001	0.918
		HIP	66	Random	1.19 (1.08–1.32)	<0.001	41.0	<0.001	0.651
	Recessive	H & HIP	109	Random	1.37 (1.23–1.52)	<0.001	43.7	<0.001	0.072
		H	35	Random	1.43 (1.21–1.68)	<0.001	45.6	0.002	0.123
		HIP	74	Random	1.34 (1.16–1.53)	<0.001	43.5	<0.001	0.118
A1298C									
	Allele contrast	H & HIP	20	Fixed	1.01 (0.92–1.11)	0.791	29.2	0.108	0.112
		H	7	Random	1.05 (0.79–1.39)	0.733	67.6	0.005	0.614
		HIP	13	Fixed	1.01 (0.90–1.14)	0.824	0.0	0.760	0.315
	Homozygous	H & HIP	20	Fixed	1.06 (0.85–1.32)	0.630	0.0	0.696	0.348
	codominant	H	7	Fixed	1.08 (0.78–1.50)	0.649	0.0	0.658	0.735
		HIP	13	Fixed	1.04 (0.77–1.40)	0.816	0.0	0.506	0.716
	Heterozygous	H & HIP	20	Fixed	0.99 (0.84–1.17)	0.928	35.4	0.060	0.818
	codominant	H	7	Random	0.96 (0.65–1.44)	0.854	71.0	0.002	0.708
		HIP	13	Fixed	1.01 (0.86–1.19)	0.918	0.0	0.760	0.716
	Dominant	H & HIP	21	Fixed	1.06 (0.90–1.26)	0.474	45.3	0.013	0.643
		H	8	Random	1.10(0.75–1.61)	0.637	77.2	<0.001	0.941
		HIP	13	Fixed	1.01 (0.87–1.18)	0.906	0.0	0.092	0.219
	Recessive	H & HIP	20	Fixed	1.10 (0.89–1.36)	0.392	0.0	0.709	0.621
		H	7	Fixed	1.15 (0.84–1.57)	0.393	0.0	0.780	0.866
		HIP	13	Fixed	1.06 (0.79–1.41)	0.712	0.0	0.453	0.528

Abbreviation: *MTHFR*, methylenetetrahydrofolate reductase; H, hypertension; HIP, hypertension in pregnancy; OR, odds ratio; CI, confidence interval; *P*_z_, *P* value for association test; *P*_h_, *P* value for heterogeneity test; *P*_e_, *P* value for publication bias test; n, the number of studies.

**Table 2:** Stratified analysis of the associations of *MTHFR* C677T polymorphism with H and HIP under dominant model.

	H & HIP			H			HIP		
Subgroup analysis	n	OR (95% CI)	*P*_h_ (I^2%^)	n	OR (95% CI)	*P*_h_ (I^2%^)	n	OR (95% CI)	*P*_h_ (I^2%^)
All HWE	81	1.23 (1.13–1.34)	<0.001 (44.9)	28	1.35 (1.17–1.54)	<0.001 (51.0)	53	1.16 (1.05–1.28)	0.003 (38.7)
**Ethnicity**									
East Asians	40	1.46 (1.29–1.66)	<0.001 (55.7)	23	1.38 (1.19–1.60)	<0.001 (55.5)	17	1.64 (1.28–2.10)	0.002 (57.6)
Caucasians	43	1.18 (1.07–1.29)	0.116 (21.0)	10	1.29 (1.01–1.63)	0.004 (62.5)	33	1.15 (1.05–1.26)	0.648 (0.00)
Latinos	10	1.09 (0.86–1.39)	0.098 (38.9)	1	1.43 (0.81–2.51)	–	9	1.06 (0.82–1.37)	0.094(41.0)
Indians and	5	0.93 (0.56–1.56)	0.004 (74.4)	1	1.71 (1.00–2.91)	–	4	0.78 (0.48–1.29)	0.039 (64.2)
Sri Lankans									
Black Africans	3	1.22 (0.89–1.67)	0.513 (0.00)	0	–	–	3	1.22 (0.89–1.67)	0.513 (0.0)
**Source of controls**									
Hospital based	80	1.30 (1.18–1.43)	<0.001 (51.0)	23	1.51 (1.30–1.75)	0.003 (50.5)	57	1.20 (1.07–1.35)	<0.001 (46.8)
Population based	21	1.19 (1.06–1.34)	0.048 (36.4)	12	1.15 (0.95–1.40)	0.005 (59.3)	9	1.22 (1.08–1.42)	0.808 (0.0)
**Genotyping methods**									
PCR-RFLP	86	1.28 (1.18–1.40)	<0.001 (50.4)	31	1.36 (1.20–1.54)	<0.001 (57.6)	55	1.23 (1.10–1.38)	<0.001 (44.5)
Others	15	1.16 (1.02–1.32)	0.122 (30.9)	4	1.40 (1.01–1.96)	0.213 (33.2)	11	1.08(0.93–1.26)	0.262 (19.1)
**Sample size**									
Large (≥225)	51	1.17 (1.07–1.27)	<0.001 (54.0)	25	1.30 (1.14–1.48)	<0.001 (58.6)	26	1.05 (0.94–1.17)	0.033 (36.7)
Small (<225)	50	1.46 (1.28–1.67)	0.014 (33.2)	10	1.63 (1.31–2.04)	0.154 (33.0)	40	1.41 (1.21–1.63)	0.025 (32.8)
**Study quality**									
High (≥5 scores)	89	1.25 (1.15–1.36)	<0.001 (49.2)	31	1.34 (1.18–1.52)	<0.001 (55.9)	58	1.19 (1.07–1.32)	<0.001 (42.8)
Low (<5 scores)	12	1.34 (1.06–1.70)	0.063 (41.8)	4	1.58 (1.03–2.42)	0.086 (54.6)	8	1.23 (1.01–1.64)	0.135 (36.8)

Abbreviation: *MTHFR*, methylenetetrahydrofolate reductase; HWE, Hardy-Weinberg equilibrium; H, hypertension; HIP, hypertension in pregnancy; OR, odds ratio; CI, confidence interval; *P*_h_, *P* value for heterogeneity test; n, the number of studies; PCR-RFLP, polymerase chain reaction-restriction fragment length polymorphism.

#### Association of *MTHFR* A1298C polymorphism with H & HIP

Twenty one studies with 2533 cases and 2976 controls on the relationship between the A1298C polymorphism and H & HIP were included in the meta-analysis. The dominant model was determined according to the principle of genetic model selection [19], [20]. No significant relationship was observed between the *MTHFR* A1298C polymorphism and H & HIP under all genetic models (Table 1 and Figure S6–S10). For the dominant model, the overall pooled OR using random effects model was 1.06 (95% CI = 0.90–1.26) (Table 1 and Figure S8). Similarly, stratified analyses based on ethnicity, source of controls, genotyping method, sample size and study quality did not reveal any significant association of the polymorphism with H & HIP (Table 3). Significant heterogeneity was observed, and meta-regression analysis was performed to explore the sources of heterogeneity. However, the H type (*P* = 0.155), year of publication (*P* = 0.351), ethnicity (*P* = 0.411), source of controls (*P* = 0.906), genotyping method (*P* = 0.197) and sample size (*P* = 0.850) were not the sources of heterogeneity.

**Table 3:** Stratified analysis of the associations of *MTHFR* A1298C polymorphism with H and HIP under dominant model.

	H & HIP			H			HIP		
Subgroup analysis	n	OR (95% CI)	*P*_h_ (I^2%^)	n	OR (95% CI)	*P*_h_ (I^2%^)	n	OR (95% CI)	*P*_h_ (I^2%^)
All HWE	18	0.96 (0.85–1.09)	0.946 (0.0)	5	0.88 (0.72–1.08)	0.698 (0.0)	13	1.01 (0.87–1.18)	0.903 (0.00)
**Ethnicity**									
East Asians	5	0.91 (0.69–1.19)	0.789 (0.00)	2	0.79 (0.56–1.12)	0.740 (0.0)	3	1.12(0.73–1.71)	0.995 (0.0)
Caucasians	12	1.00 (0.85–1.25)	0.041 (45.9)	5	1.02 (0.64–1.64)	0.003 (74.8)	7	0.91 (0.74–1.13)	0.727 (0.0)
Latinos	1	1.25 (0.44–3.56)	–	0	–	–	1	1.25 (0.44–3.56)	–
Indians and	2	1.79 (0.72–4.45)	0.011 (84.6)	1	2.91 (1.64–5.15)	–	1	1.15 (0.75–1.76)	–
Sri Lankans									
Black Africans	1	1.11 (0.78–1.59)	–	0	–	–	1	1.11 (0.78–1.59)	–
**Source of controls**									
Hospital based	14	1.16 (0.98–1.36)	0.396 (5.0)	3	1.34 (0.59–3.00)	0.006 (80.2)	11	1.09 (0.91–1.30)	0.996 (0.00)
Population based	7	0.93 (0.79–1.10)	0.003 (69.4)	5	0.98 (0.62–1.55)	<0.001 (77.4)	2	0.79 (0.57–1.08)	0.516 (0.00)
**Genotyping methods**									
PCR-RFLP	18	1.03 (0.87–1.21)	0.065 (36.0)	7	1.00 (0.67–1.47)	<0.001 (72.5)	11	1.03 (0.87–1.21)	0.926 (0.00)
Others	3	1.25 (0.67–2.33)	0.026 (72.5)	1	1.98 (1.28–3.08)	–	2	0.88 (0.57–1.37)	0.396(0.00)
**Sample size**									
Large (≥170)	10	1.10 (0.86–1.40)	<0.001 (69.6)	5	1.26 (0.78–2.03)	<0.001 (83.2)	5	0.97 (0.80–1.17)	0.331 (13.0)
Small (<170)	11	1.02 (0.81–1.29)	0.735 (0.0)	3	0.81 (0.39–1.07)	0.070 (62.5)	8	1.11 (0.84–1.47)	1.000 (0.00)
**Study quality**									
High (≥5 scores)	19	1.09 (0.92–1.29)	0.030 (41.6)	7	1.22 (0.84–1.79)	<0.001 (75.8)	12	0.99 (0.84–1.17)	0.928 (0.00)
Low (<5 scores)	2	0.72 (0.24–2.16)	0.023 (80.6)	1	0.39 (0.17–0.89)	–	1	1.20 (0.72–2.00)	–

Abbreviation: *MTHFR*, methylenetetrahydrofolate reductase; HWE, Hardy-Weinberg equilibrium; H, hypertension; HIP, hypertension in pregnancy; OR, odds ratio; CI, confidence interval; *P*_h_, *P* value for heterogeneity test; n, the number of studies; PCR-RFLP, polymerase chain reaction-restriction fragment length polymorphism.

#### Association of *MTHFR* C677T polymorphism with H

Thirty six studies with 6584 cases and 6760 controls reporting the relationship between the *MTHFR* C677T polymorphism and H were included in our meta-analysis. The results of overall pooled analyses under five genetic models are listed in Table 1. The dominant model was determined according to the principle of genetic model selection [19], [20]. The summary results indicated that the polymorphism was significantly associated with H. For the dominant model, the overall pooled OR using random effects model was 1.36 (95% CI = 1.20–1.53). Table 2 summarizes the results of stratified analyses under dominant genetic model. As stratified analysis by ethnicity, significant associations were found among East Asians and Caucasians, but not among Latinos, Black Africans, and Indians and Sri Lankans. Stratified analysis by source of controls showed significant association in hospital based studies, but not in population based studies. When stratified analyses were conducted based on genotyping method, sample size and study quality, significant associations were found in all the subgroups. Meta-regression was performed to find the sources of heterogeneity. However, the year of publication (*P* = 0.191), ethnicity (*P* = 0.953), source of controls (*P* = 0.066), genotyping method (*P* = 0.734) and sample size (*P* = 0.551) were not the sources of heterogeneity.

#### Association of *MTHFR* A1298C polymorphism with H

We identified eight studies with 1196 cases and 1213 controls investigating the relationship of the polymorphism with H. The results of overall pooled analyses under five genetic models are listed in Table 1. The dominant model was determined according to the principle of genetic model selection [19], [20]. In the overall comparison, the polymorphism was not significantly with H in any of the genetic model. For the dominant model, the overall pooled OR using random effects model was 1.10 (95% CI = 0.75–1.61) (Table 1). As stratified analyses by ethnicity, genotyping method and study quality, significant associations were found in Indians and Sri Lankans and “others” genotyping method studies, whereas a significant negative association was found in low quality studies (Table 3). Notably, each of these associations was based on only one study; therefore, the results should be interpreted with great caution (Table 3). Although significant heterogeneity existed, a meta-regression analysis was not performed due to the limited number of the studies (<10) included in this group.

#### Association of *MTHFR* C677T polymorphism with HIP

Seventy five studies with 8510 cases and 14873 controls on the relationship between the *MTHFR* C677T polymorphism and HIP were included in the meta-analysis. The results of overall pooled analyses under five genetic models are presented in Table 1. The dominant model was determined according to the principle of genetic model selection [19], [20]. The summary results indicated that the polymorphism was significantly associated with HIP. For the dominant model, the overall pooled OR using random effects model was 1.19 (95% CI = 1.08–1.32) (Table 1). Results from subgroup analysis based on ethnicity indicated that the C677T polymorphism was associated with HIP among East Asians and Caucasians. However, no significant associations were found among Latinos, Black Africans, and Indians and Sri Lankans. As stratified analyses by source of controls, genotyping method, sample size and study quality, significant associations were found in all the subgroups, with the exception of large sample size subgroup and “others” genotyping method subgroup (Table 2). To explore the sources of heterogeneity, a meta-regression was performed, and the results showed that ethnicity had a statistical significance (*P = *0.004) while the year of publication (*P* = 0.240), source of controls (*P* = 0.290), genotyping method (*P* = 0.476) and sample size (*P* = 0.713) had no statistical significance.

#### Association of *MTHFR* A1298C polymorphism with HIP

Thirteen studies with 1337 cases and 1763 controls on the relationship of the *MTHFR* A1298C polymorphism with HIP were included in the meta-analysis. The summary results of overall pooled analysis under five genetic models are showed in Table 1. The dominant model was determined according to the principle of genetic model selection [19], [20]. The summary results indicated that the polymorphism was not significantly associated with HIP. For the dominant model, the pooled OR using fixed effects model was 1.01 (95% CI = 0.87–1.18) (Table 1). Similarly, in the stratified analyses by ethnicity, source of controls, genotyping method, sample size and study quality, no significant association was found in all the subgroups (Table 3).

### Cumulative Meta-analysis

Cumulative meta-analyses were performed using a dominant model for the *MTHFR* C677T and A1298C polymorphisms. Regarding to C677T, a trend of a more significant association was consistently observed with a narrowing of the 95% CI as information accumulated by year (Figure S11). However, for A1298C, as studies were published, the association of the polymorphism with H & HIP was statistically non-significant (Figure S12).

### Sensitivity Analysis

Sensitivity analysis was performed to confirm the stability and liability of the meta-analysis by sequentially omitting individual eligible studies. When any single study was excluded, the corresponding ORs were not materially changed (data were not shown), indicating the stability of our results. Additionally, we excluded the studies that genotype distribution in the controls deviating from HWE, and the corresponding pooled ORs were not significantly changed (Table 2 and Table 3).

### Publication Bias

**Figure 3:**
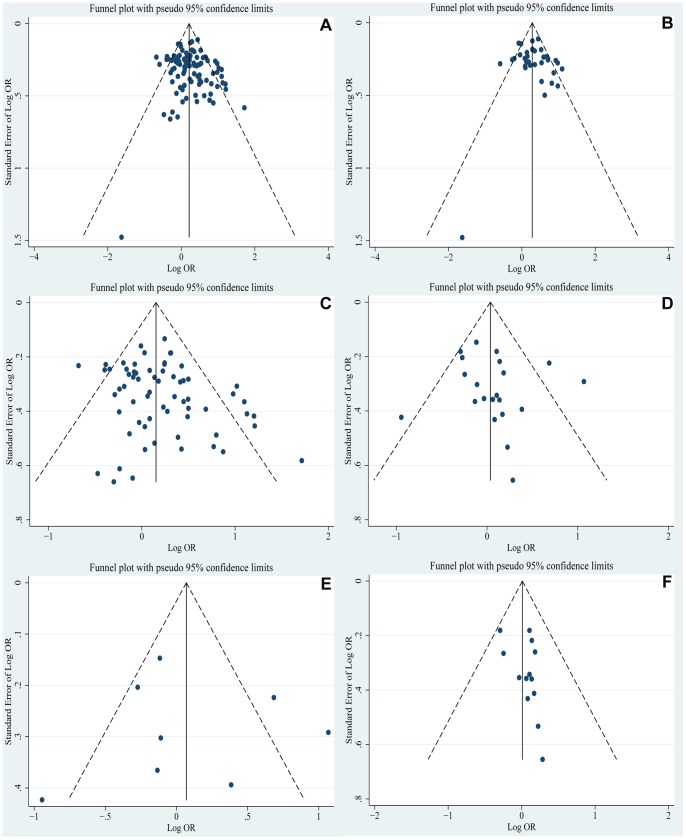
Funnel plot analysis on the detection of publication bias in the meta-analysis of the associations between *MTHFR* polymorphisms and H & HIP (A: C677T and H & HIP; B: C677T and H; C: C677T and HIP; D: A1298C and H & HIP; E: A1298C and H; F: A1298C and HIP).

Funnel plot and Egger’s linear regression were performed to assess the publication bias of the included studies. The shapes of the funnel plots did not reveal any evidence of obvious asymmetry (Figure 3). The results of Egger’s test also showed that there was no strong statistical evidence of publication bias (Table 1).

## Discussion

The present meta-analysis involved 111 studies with 15094 cases and 21633 controls that investigated the C677T polymorphism and 21 studies with 2533 cases and 2976 controls investigated the A1298C polymorphism. Overall, our meta-analytical results provided evidences that the *MTHFR* C677T polymorphism was associated with both H and HIP (H & HIP: OR = 1.26, 95% CI = 1.17–1.34; H: OR = 1.36, 95% CI = 1.20–1.53; HIP: OR = 1.19, 95% CI = 1.08–1.32). However, no association was detected between the *MTHFR* A1298C polymorphism and H & HIP (H & HIP: OR = 1.06, 95% CI = 0.90–1.26; H: OR = 1.10, 95% CI = 0.75–1.61; HIP: OR = 1.01, 95% CI = 0.87–1.18). Sensitivity analysis and cumulative meta-analysis further strengthened the validity of these results.

In recent years several meta-analyses have been done to investigate the associations of the *MTHFR* C677T polymorphism with H and/or HIP, and our findings were largely in line with these published meta-analyses [16], [17], [139]–[141]. Intuitively, our study is seemingly superfluous, but it enjoyed apparent superiority over these previous meta-analyses in terms of the following aspects: first, we performed literature searches from seven electronic databases including PubMed, Embase, Web of Science, CNKI, Wanfang, CBM and VIP, while these previous meta-analyses only searched part of the aforementioned databases, thus our more comprehensive search can ensure as many studies as possible and minimize selection bias; second, our study inspected not only H but HIP and included approximately 6 times as many participants as Niu et al. [17], Qian et al. [16] and Kosmas et al.’s [139] meta-analyses, and 23 more studies than the most recent meta-analysis by Wang et al. [141]; finally, besides stratified analyses, we further performed meta-regression and cumulative meta-analysis to investigate potential sources of heterogeneity and study stability respectively. Based on the above advantages, our study can provide a more precise estimation of associations between the C677T polymorphism and H & HIP.

After subgroup analysis according to ethnicity, the results indicated that the *MTHFR* C677T polymorphism was associated with H & HIP among East Asians and Caucasians, but not among Latinos, Black Africans, and Indians and Sri Lankans. Several factors may contribute to the phenomenon that the C677T polymorphism was associated with H & HIP in one population and the association was nil for another population. Above all, different genetic backgrounds may attribute to the discrepancy, since the 677T allele distributions vary among Latinos, East Asians, Caucasians, Black Africans, and Indians and Sri Lankans, with a prevalence of 41.1%, 32.5%, 30.6%, 12.3% and 6.7%, respectively. Another explanation may be that different populations live with multiple life styles and environmental factors, some of which may affect disease development [2]. Other factors such as selection bias and different matching criteria should also be considered. Additionally, relative small sample sizes for Latinos, Black Africans, and Indians and Sri Lankans limited us to detect stable effects in these populations. Therefore, additional studies are warranted to validate possible ethnic differences in the associations of the C677T polymorphism with H & HIP, especially among Latinos, Black Africans, and Indians and Sri Lankans. When stratifying by source of controls and sample size, significant associations were observed in almost all the subgroups, with the exception of population based subgroup in H association studies and large sample size subgroup in HIP association studies. In addition, hospital based and small sample size studies seem to have stronger associations than population based and large sample size studies. Hospital based studies are prone to produce unreliable results because controls from hospital based studies are less representative of the general population, especially when the polymorphism under investigation are expected to be related to disorders that the hospital based controls may have [142], [143]. Small sample with limited participants is often accompanied with selection biases, and lacks sufficient power to support or deny an association [144]. It is therefore speculated that our meta-analysis might overestimate the magnitude of association between the polymorphism and H & HIP in the overall effect estimates. Although this may not influence the final conclusions, further large scale and well designed population based studies are warranted to explore the associations reliably. Stratified analysis by genotyping method suggested significant associations in both PCR-RFLP and “others” genotyping method studies, except among those HIP association studies taking “others” as genotyping method. PCR-RFLP is the most commonly used method for genotyping *MTHFR* in this meta-analysis because of its relative simplicity. Although it is reported that other genotyping methods (Taqman, Mass Array and gene chip) may provide high sensitivity and accuracy in SNP genotyping under optimized condition [145], [146], [147], only 12 of total 114 studies included in our meta-analysis employed these genotyping methods. Thus the discrepancies should be concerned with great caution, and the sensitivity and specificity of those genotyping techniques should be further explored to seek out the optimal approaches that could minimize the genotyping errors.

To the best of our knowledge, this is the first comprehensive meta-analysis to date investigating the associations between the *MTHFR* A1298C polymorphism and H & HIP. Overall, our meta-analytical results indicated that the A1298C polymorphism was not associated with either H or HIP. In the stratified analyses according to ethnicity, source of controls, genotyping method, sample size and study quality, no evidence of any gene-association was obtained in almost all the subgroups. Although significant associations were found in Indians and Sri Lankans, “others” genotyping method and low quality subgroups for H association studies, these results should be interpreted with great caution because only one study was included in each of these subgroups. The overall lack of the correlation may be due to relatively small sample numbers of studies and participants. Detecting a very small effect may require much larger sample sizes. Another potential explanation may be that the effect of a single polymorphism might have a limited effect on H & HIP. This is consistent with the hypothesis that H & HIP are multi-factorial conditions that result from complicated interactions between environmental and genetic factors.

Several potential limitations of the present meta-analysis should be acknowledged. Firstly, significant heterogeneity was observed in overall and subgroup analyses, especially for the *MTHFR* C677T polymorphism. Although several potential sources of the heterogeneity were investigated including ethnicity, year of publication, source of controls, genotyping, sample size and study quality, none of them sufficiently explain the between-study heterogeneity. These results indicated that other unmeasured characteristics in various study populations and/or inherited limitations of the included studies might partially cause the detected heterogeneity. Secondly, the sample size of the *MTHFR* A1298C polymorphism involved is not large enough, especially for subgroup analysis. Thus they do not have adequate power to detect the possible association for this polymorphism and the observed significant associations in some subgroup analyses may be false. For the *MTHFR* C677T polymorphism, the results for East Asians and Black Africans should also be interpreted with caution due to the limited sample size. Thirdly, although funnel plot and Egger’s test showed that publication bias was not evident in the present study, selection bias might have occured because only studies in English and Chinese (expect one study in Persian) were included in our meta-analysis. Finally, gene-gene, gene-environment or even the different polymorphism loci of the *MTHFR* gene interactions were not estimated in our study because of the insufficient information. Despite these limitations, our meta-analysis has several clear advantages: (1) including a substantial number of cases and controls (15411 cases and 21970 controls) from different studies, thus guaranteeing the statistical power of our meta-analysis and obtaining more precise estimates; (2) the quality of the studies included in this meta-analysis was sufficient according to our well-designed selection criteria; (3) no evidence of publication bias was observed, and cumulative meta-analysis and sensitivity analysis indicated that our results were statistically robust.

In conclusion, our meta-analysis provides evidences that the *MTHFR* C677T polymorphism is associated with H & HIP, especially among East Asians and Caucasians. However, the *MTHFR* A1298C polymorphism is not associated with H & HIP. Considering the limitations aforementioned, further large-scale and population based studies, especially among Black Africans and Indians and Sri Lankans, are warranted to validate the associations observed in our meta-analysis and to explore the potential gene-gene and gene-environment interactions between the polymorphisms and H & HIP.

## Supporting Information

Figure S1 **Forest plot of the association between** ***MTHFR*** **C677T polymorphism and H & HIP in homozygous codominant model (TT vs. CC).**
(TIF)

Figure S2 **Forest plot of the association between** ***MTHFR*** **C677T polymorphism and H & HIP in heterozygous codominant model (CT vs. CC).**
(TIF)

Figure S3 **Forest plot of the association between** ***MTHFR*** **C677T polymorphism and H & HIP in dominant model (TT+CT vs. CC).**
(TIF)

Figure S4 **Forest plot of the association between** ***MTHFR*** **C677T polymorphism and H & HIP in recessive model (TT vs. CT+CC).**
(TIF)

Figure S5 **Forest plot of the association between** ***MTHFR*** **C677T polymorphism and H & HIP in allele contrast model (T vs. C).**
(TIF)

Figure S6 **Forest plot of the association between** ***MTHFR*** **A1298C polymorphism and H & HIP in homozygous codominant model (CC vs. AA).**
(TIF)

Figure S7 **Forest plot of the association between** ***MTHFR*** **A1298C polymorphism and H & HIP in heterozygous codominant model (AC vs. AA).**
(TIF)

Figure S8 **Forest plot of the association between** ***MTHFR*** **A1298C polymorphis and H & HIP in dominant model (CC+AC vs. AA).**
(TIF)

Figure S9 **Forest plot of the association between** ***MTHFR*** **A1298C polymorphism and H & HIP in recessive model (CC vs AC+AA).**
(TIF)

Figure S10 **Forest plot of the association between** ***MTHFR*** **A1298 polymorphism and H & HIP in allele contrast model (C vs A).**
(TIF)

Figure S11 **The cumulative forest plot of OR with 95% CI for** ***MTHFR*** **C677T polymorphism and H &HIP in dominant model.**
(TIF)

Figure S12 **The cumulative forest plot of OR with 95% CI for** ***MTHFR*** **A1298C polymorphism and H & HIP in dominant model.**
(TIF)

Table S1 **Baseline characteristics of qualified studies in this meta-analysis.**
(DOC)

Table S2 **Distribution of genotype and allele frequencies of the** ***MTHFR*** **C677T polymorphism.**
(DOC)

Table S3 **Distribution of genotype and allele frequencies of the** ***MTHFR*** **A1298C polymorphism.**
(DOC)

Check list S1 **PRISMA checklist.**
(DOC)
