# Supplementary figures and images for: Associations of MTHFR Gene Polymorphisms with Hypertension and Hypertension in Pregnancy: A Meta-Analysis from 114 Studies with 15411 Cases and 21970 Controls
Source: PLoS One. 2014 Feb 5;9(2):e87497. doi: 10.1371/journal.pone.0087497 (PMC3914818; doi:10.1371/journal.pone.0087497)

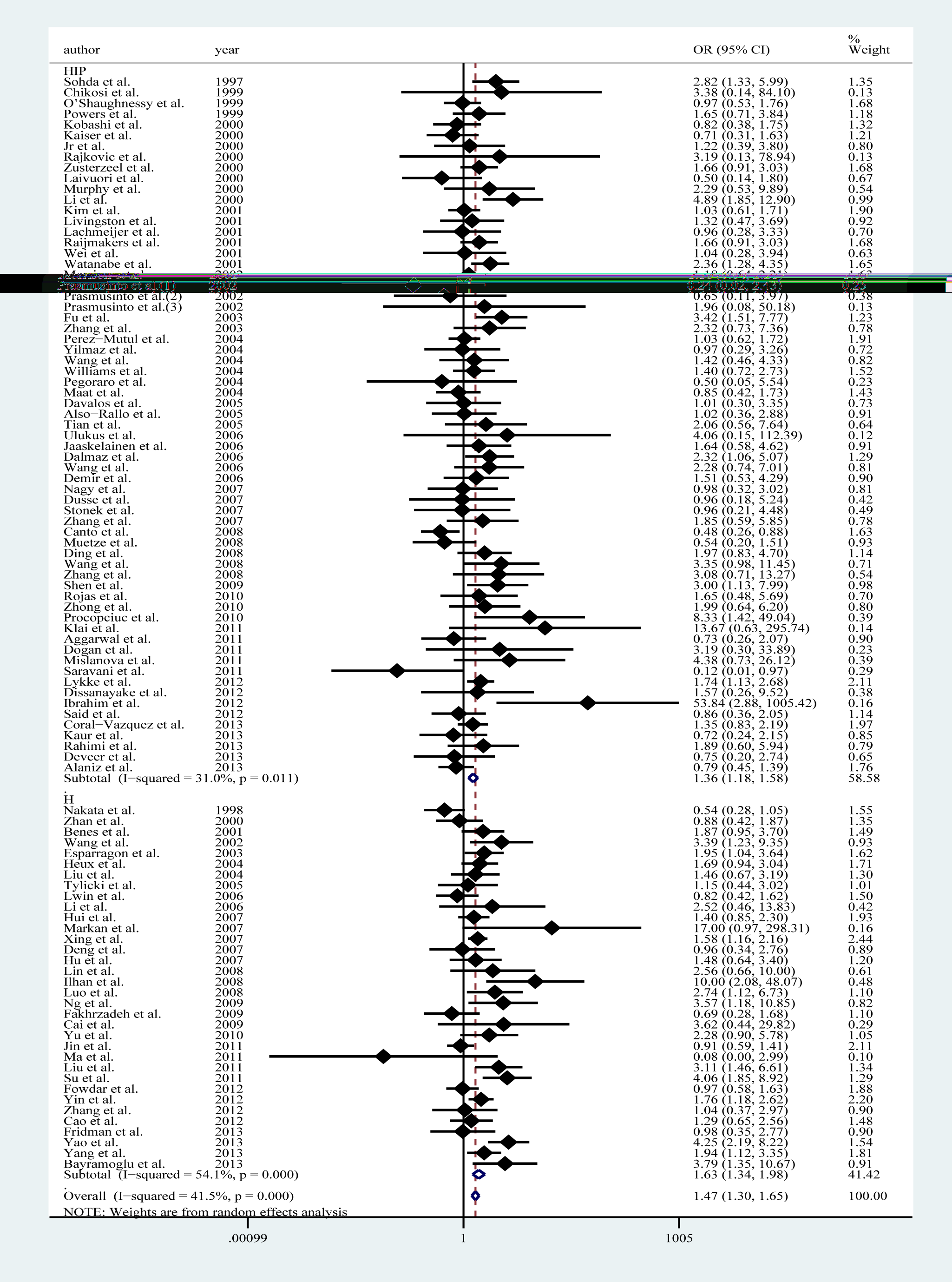

Supplement: Figure S1 — Forest plot of the association between MTHFR C677T polymorphism and H & HIP in homozygous codominant model (TT vs. CC). (TIF) [file pone.0087497.s001.tif]

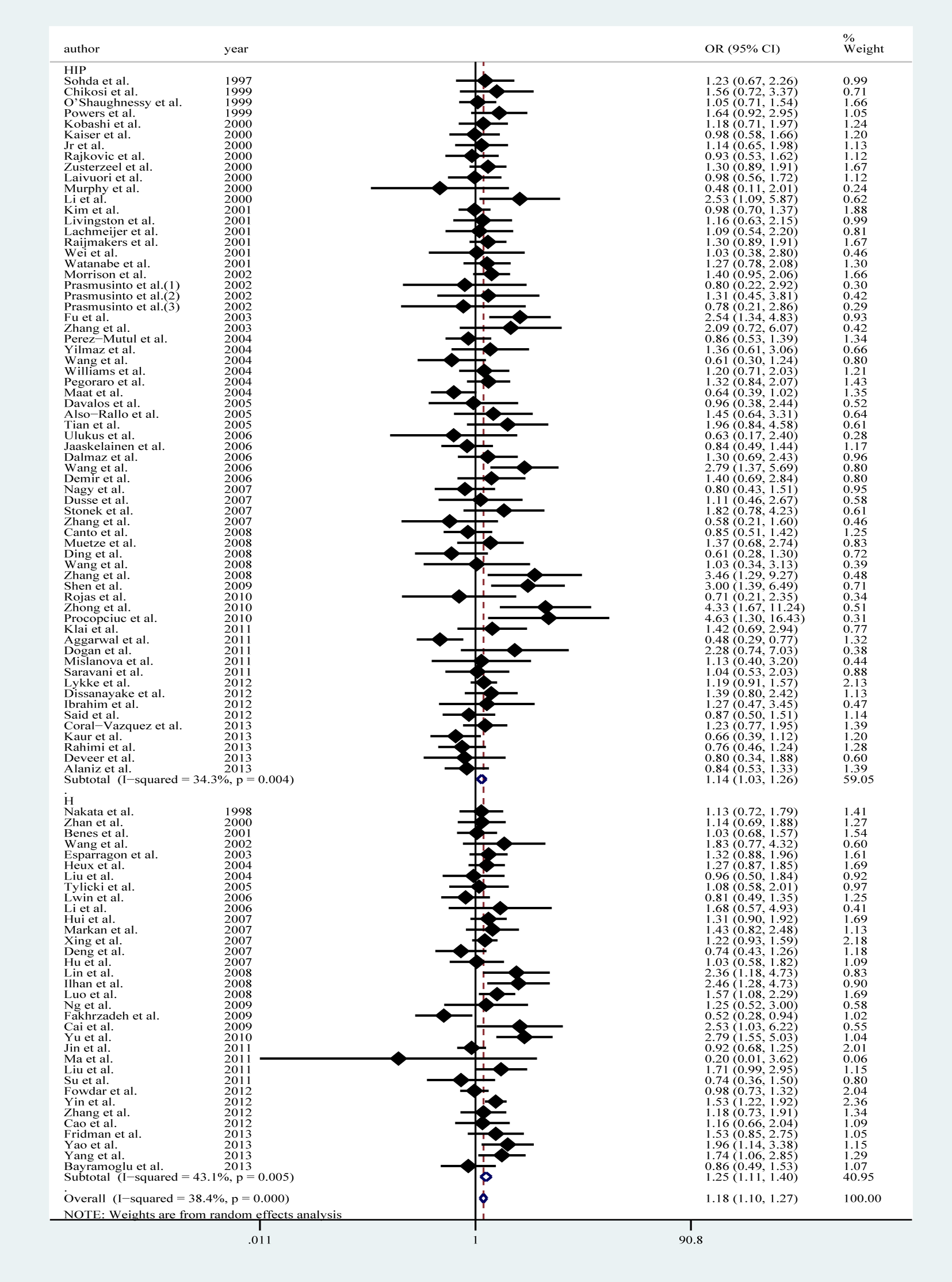

Supplement: Figure S2 — Forest plot of the association between MTHFR C677T polymorphism and H & HIP in heterozygous codominant model (CT vs. CC). (TIF) [file pone.0087497.s002.tif]

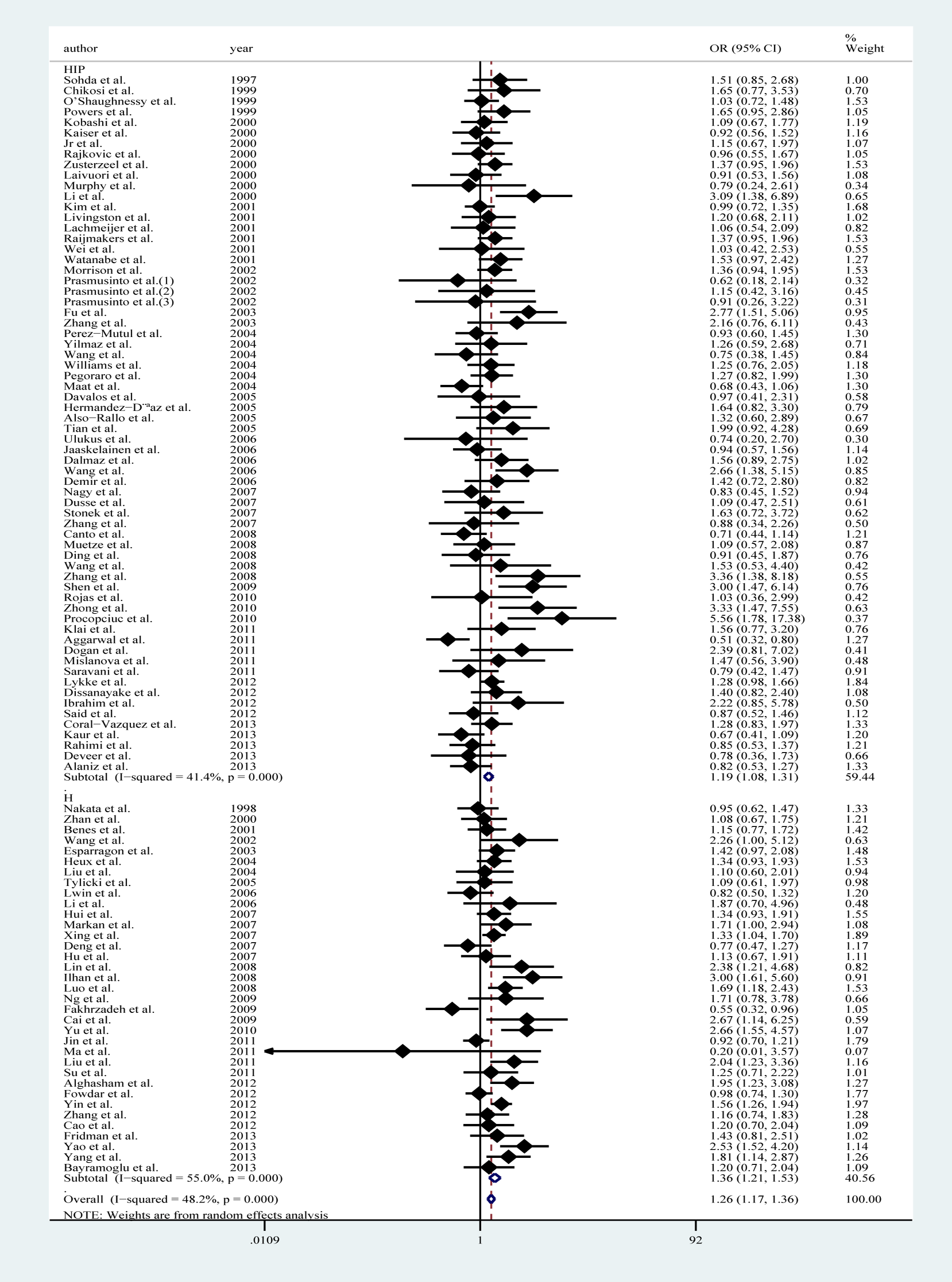

Supplement: Figure S3 — Forest plot of the association between MTHFR C677T polymorphism and H & HIP in dominant model (TT+CT vs. CC). (TIF) [file pone.0087497.s003.tif]

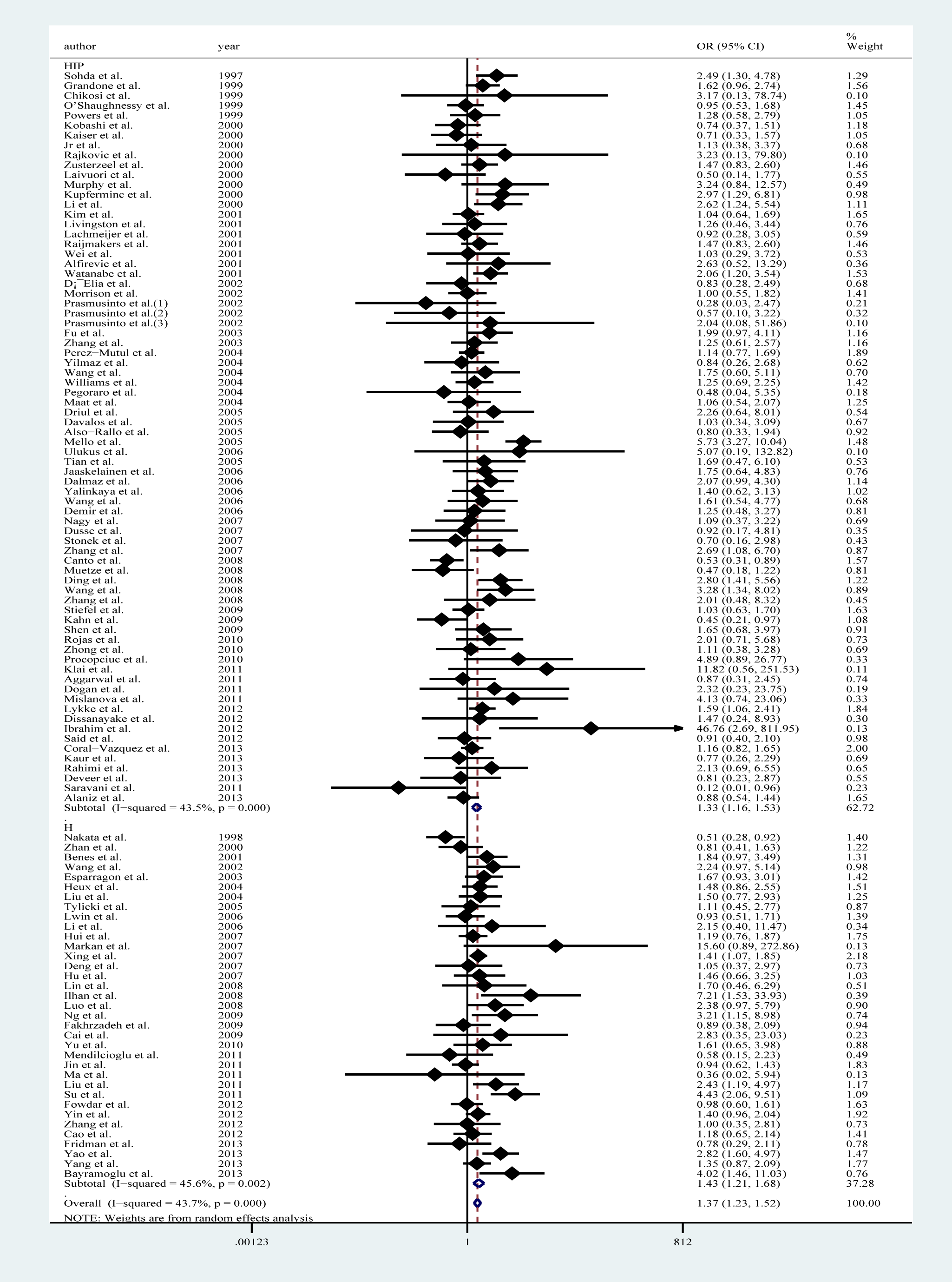

Supplement: Figure S4 — Forest plot of the association between MTHFR C677T polymorphism and H & HIP in recessive model (TT vs. CT+CC). (TIF) [file pone.0087497.s004.tif]

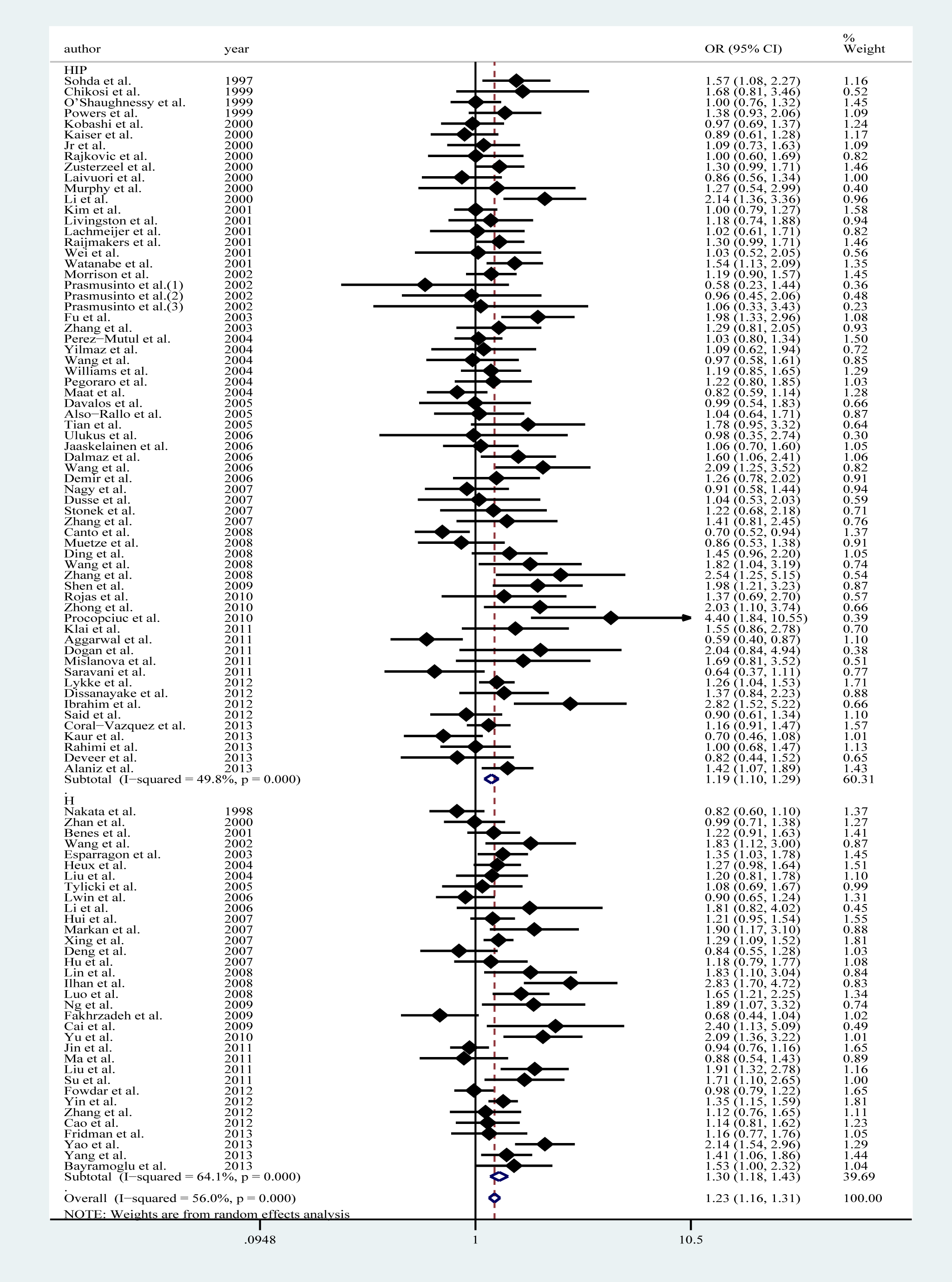

Supplement: Figure S5 — Forest plot of the association between MTHFR C677T polymorphism and H & HIP in allele contrast model (T vs. C). (TIF) [file pone.0087497.s005.tif]

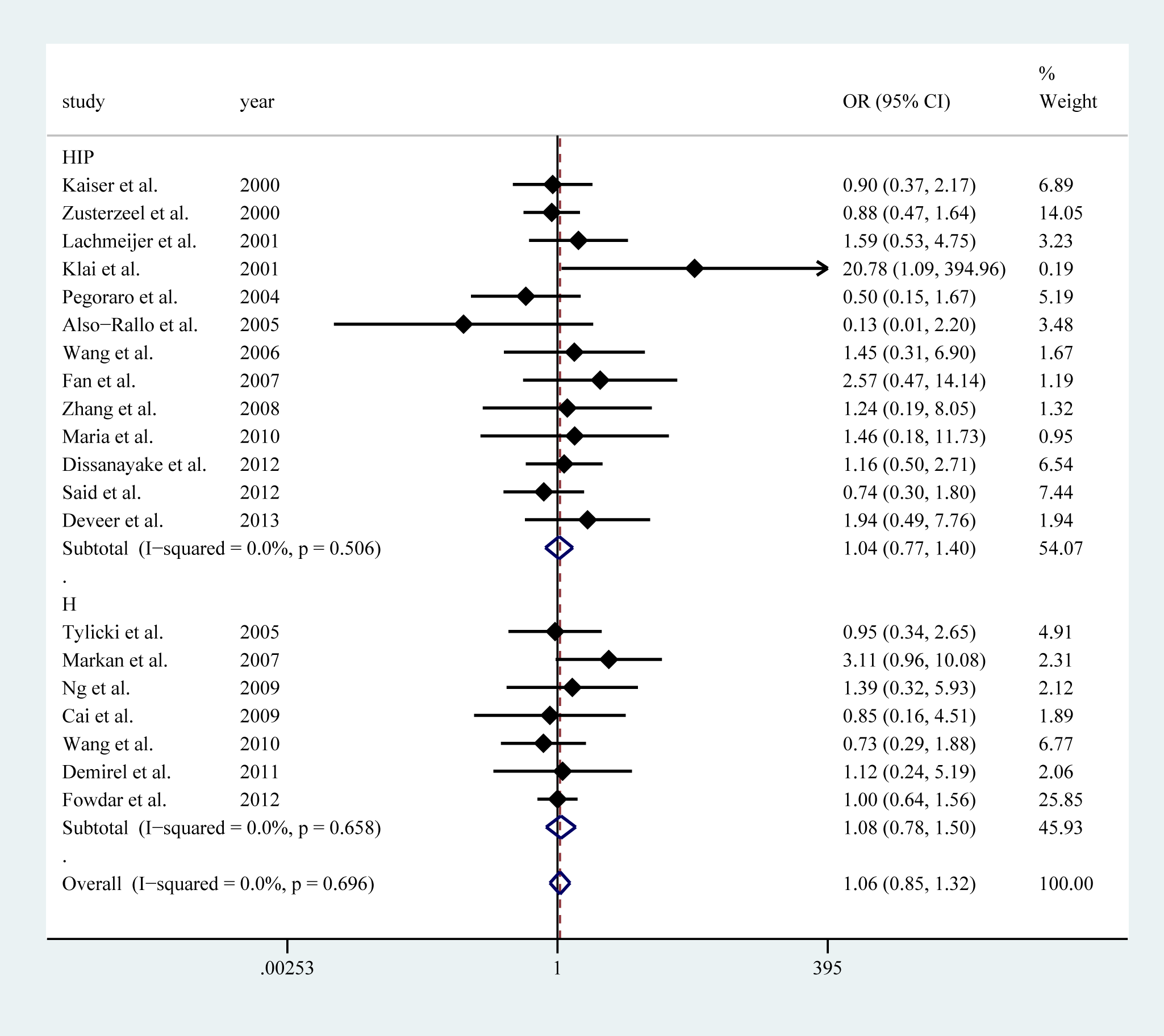

Supplement: Figure S6 — Forest plot of the association between MTHFR A1298C polymorphism and H & HIP in homozygous codominant model (CC vs. AA). (TIF) [file pone.0087497.s006.tif]

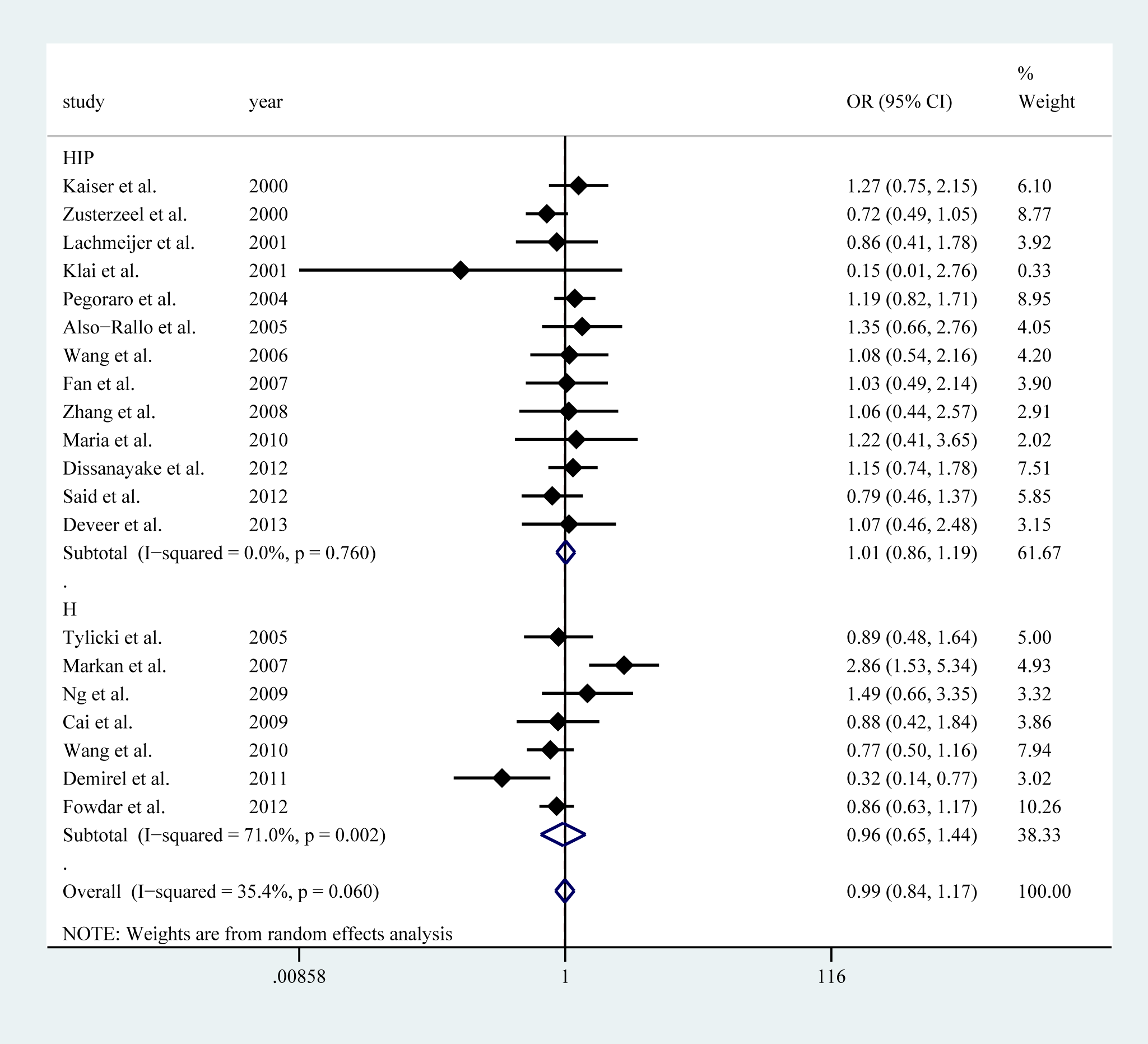

Supplement: Figure S7 — Forest plot of the association between MTHFR A1298C polymorphism and H & HIP in heterozygous codominant model (AC vs. AA). (TIF) [file pone.0087497.s007.tif]

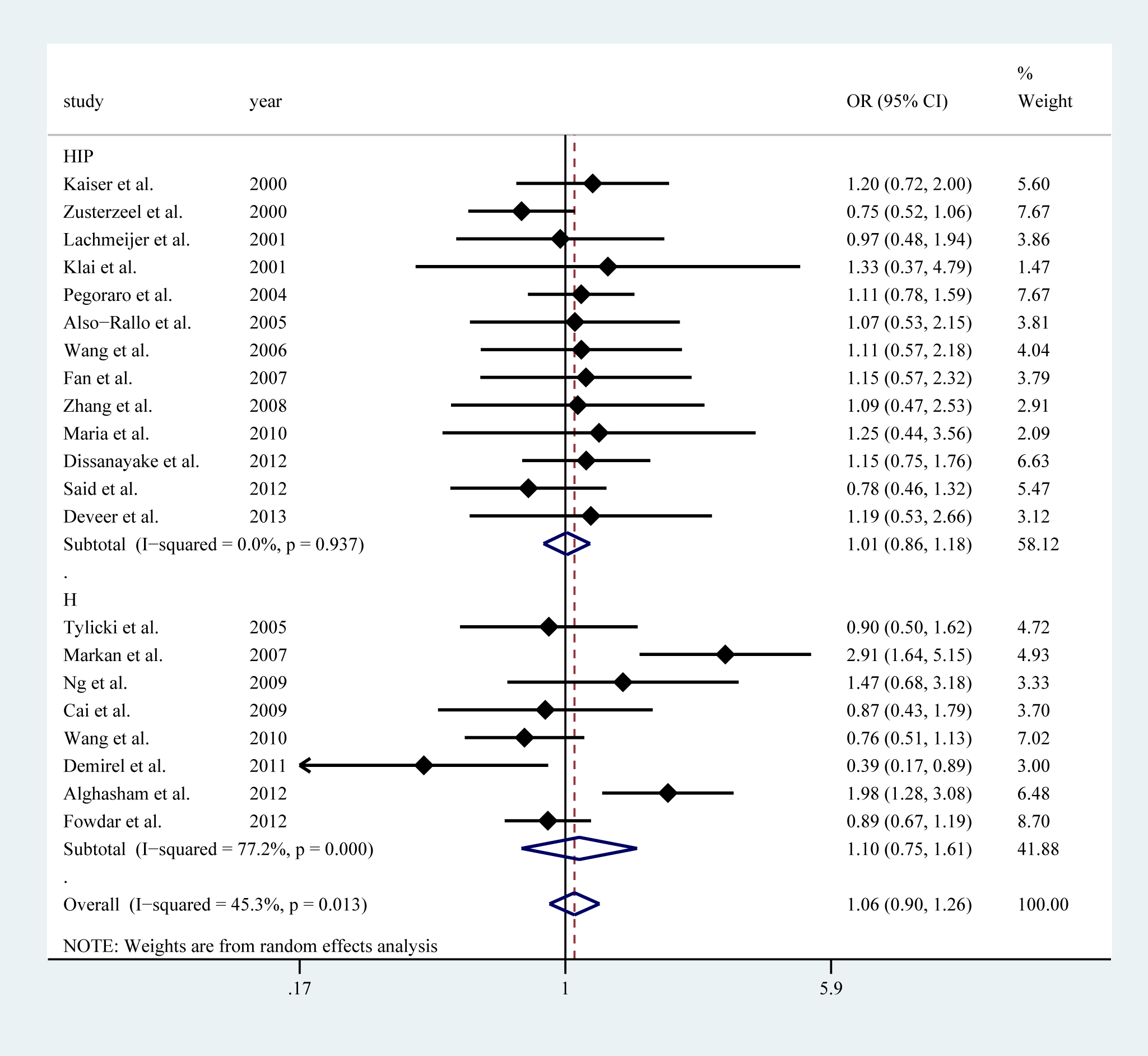

Supplement: Figure S8 — Forest plot of the association between MTHFR A1298C polymorphis and H & HIP in dominant model (CC+AC vs. AA). (TIF) [file pone.0087497.s008.tif]

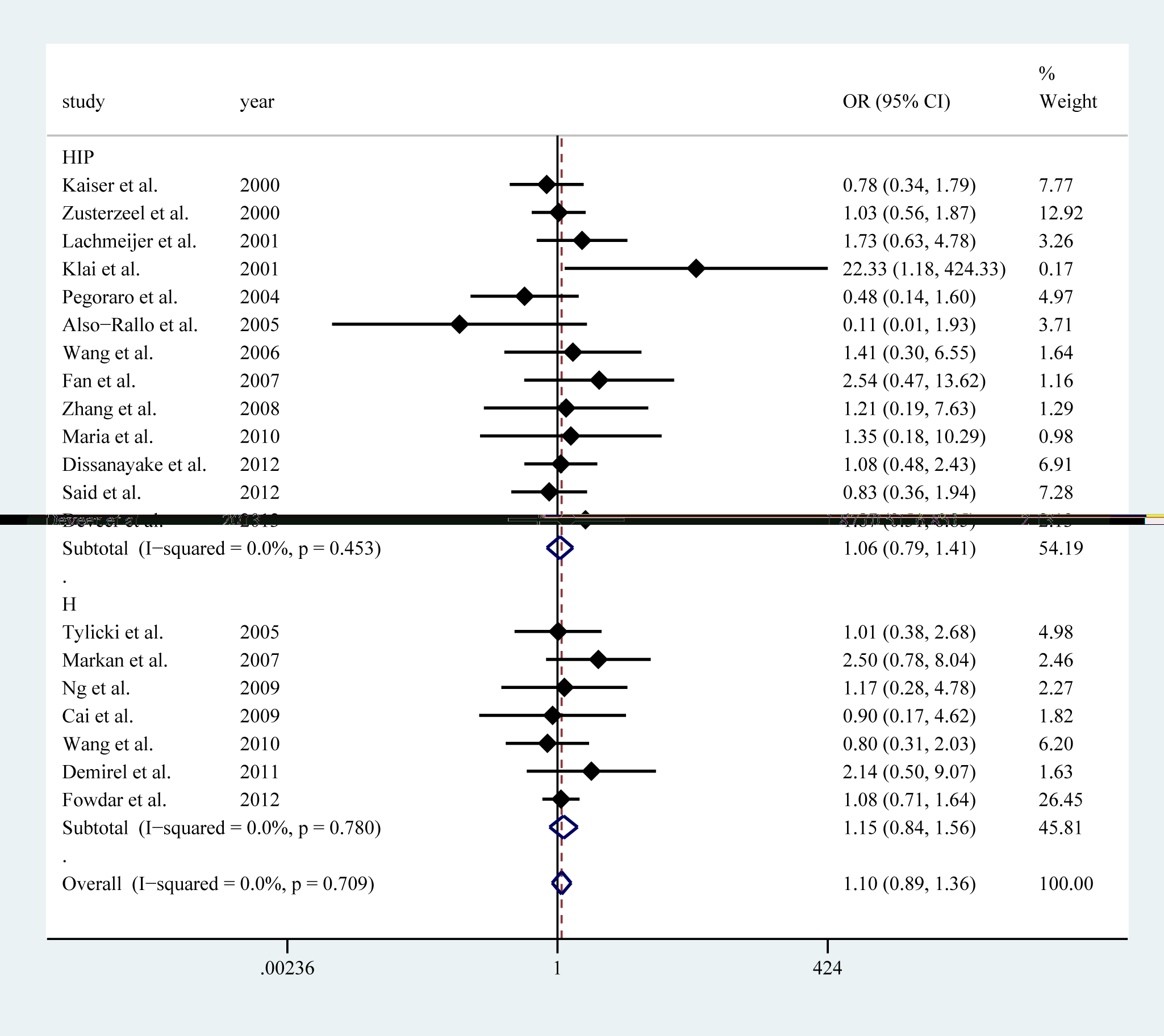

Supplement: Figure S9 — Forest plot of the association between MTHFR A1298C polymorphism and H & HIP in recessive model (CC vs AC+AA). (TIF) [file pone.0087497.s009.tif]

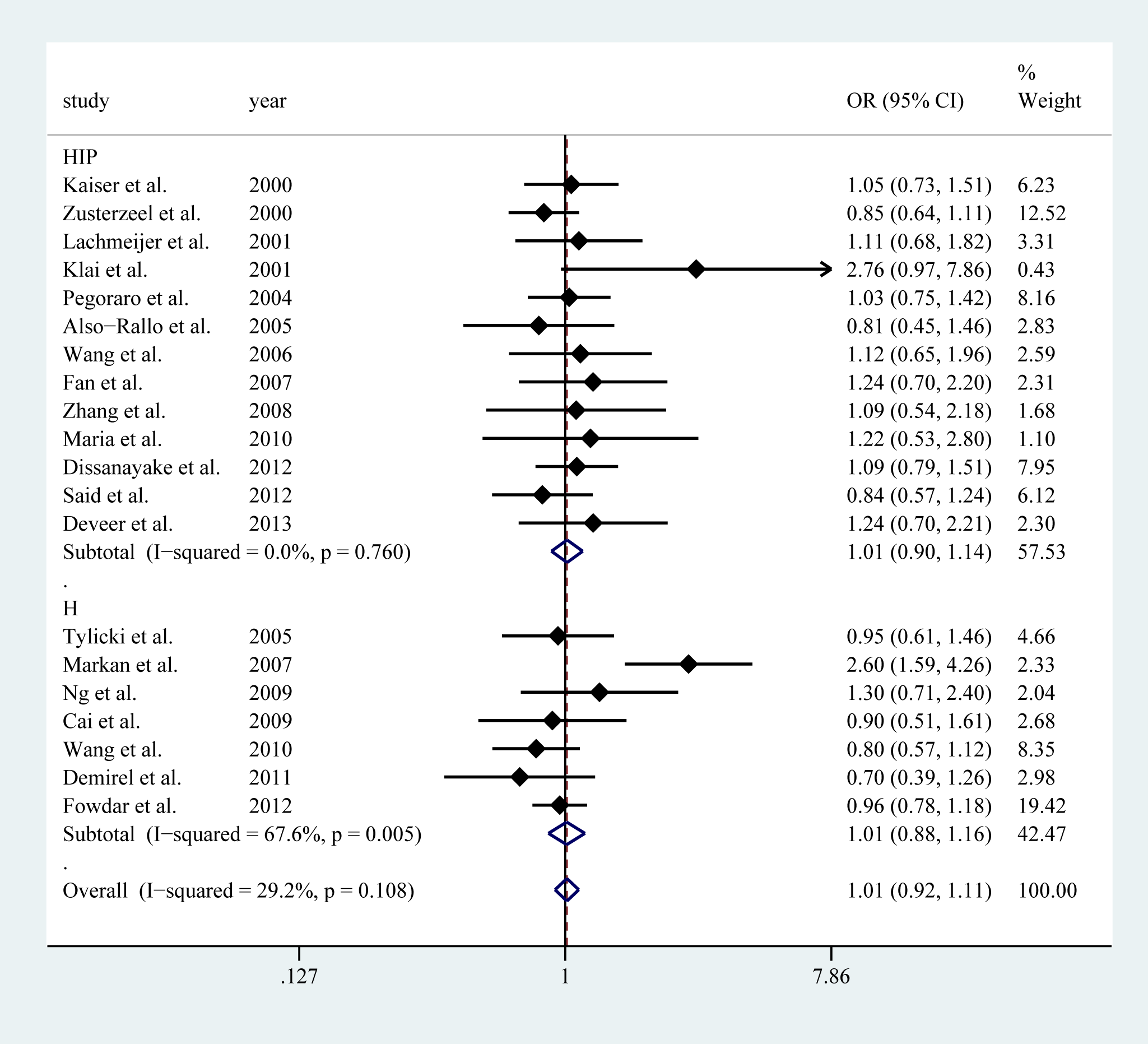

Supplement: Figure S10 — Forest plot of the association between MTHFR A1298 polymorphism and H & HIP in allele contrast model (C vs A). (TIF) [file pone.0087497.s010.tif]

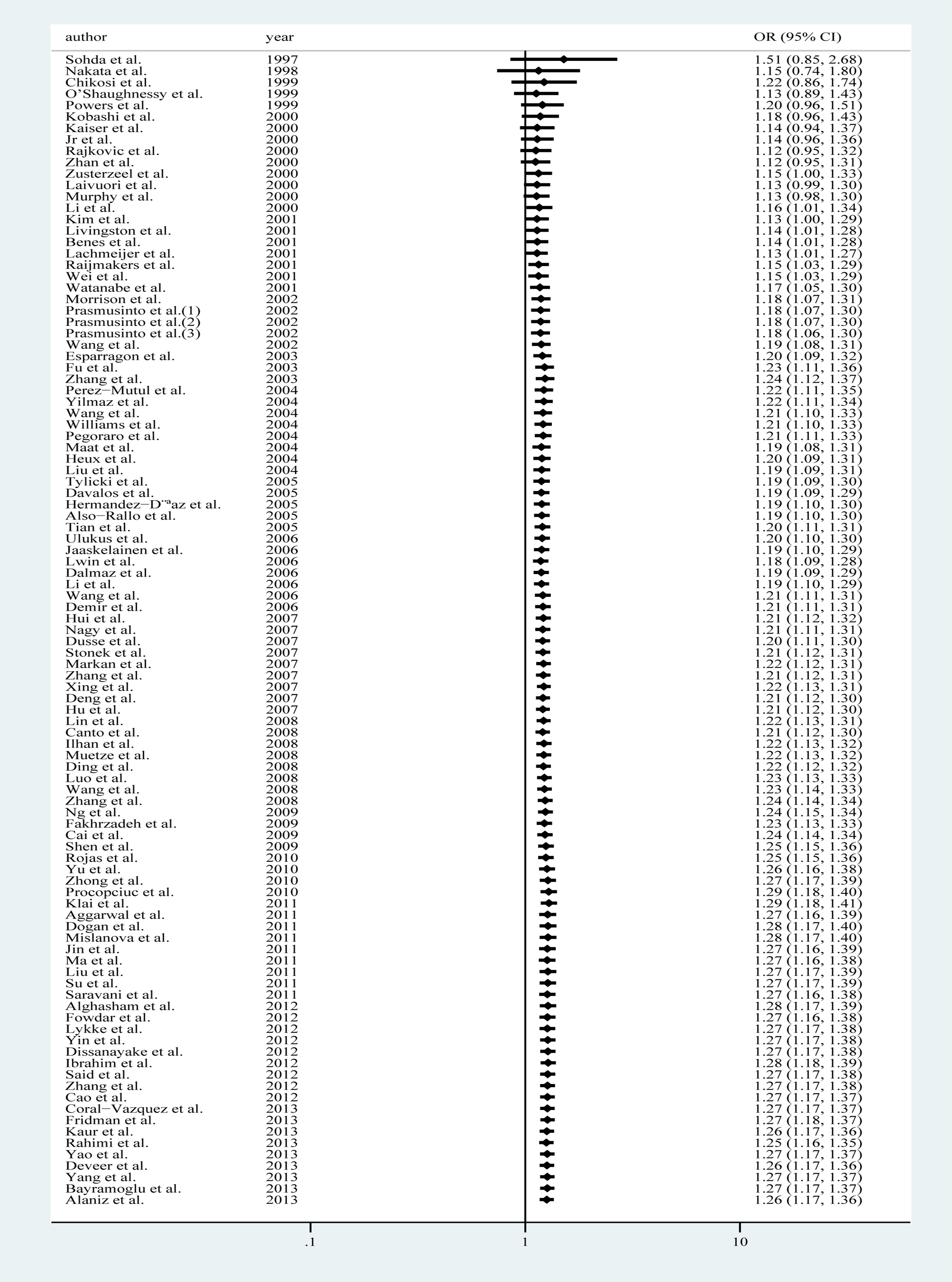

Supplement: Figure S11 — The cumulative forest plot of OR with 95% CI for MTHFR C677T polymorphism and H &HIP in dominant model. (TIF) [file pone.0087497.s011.tif]

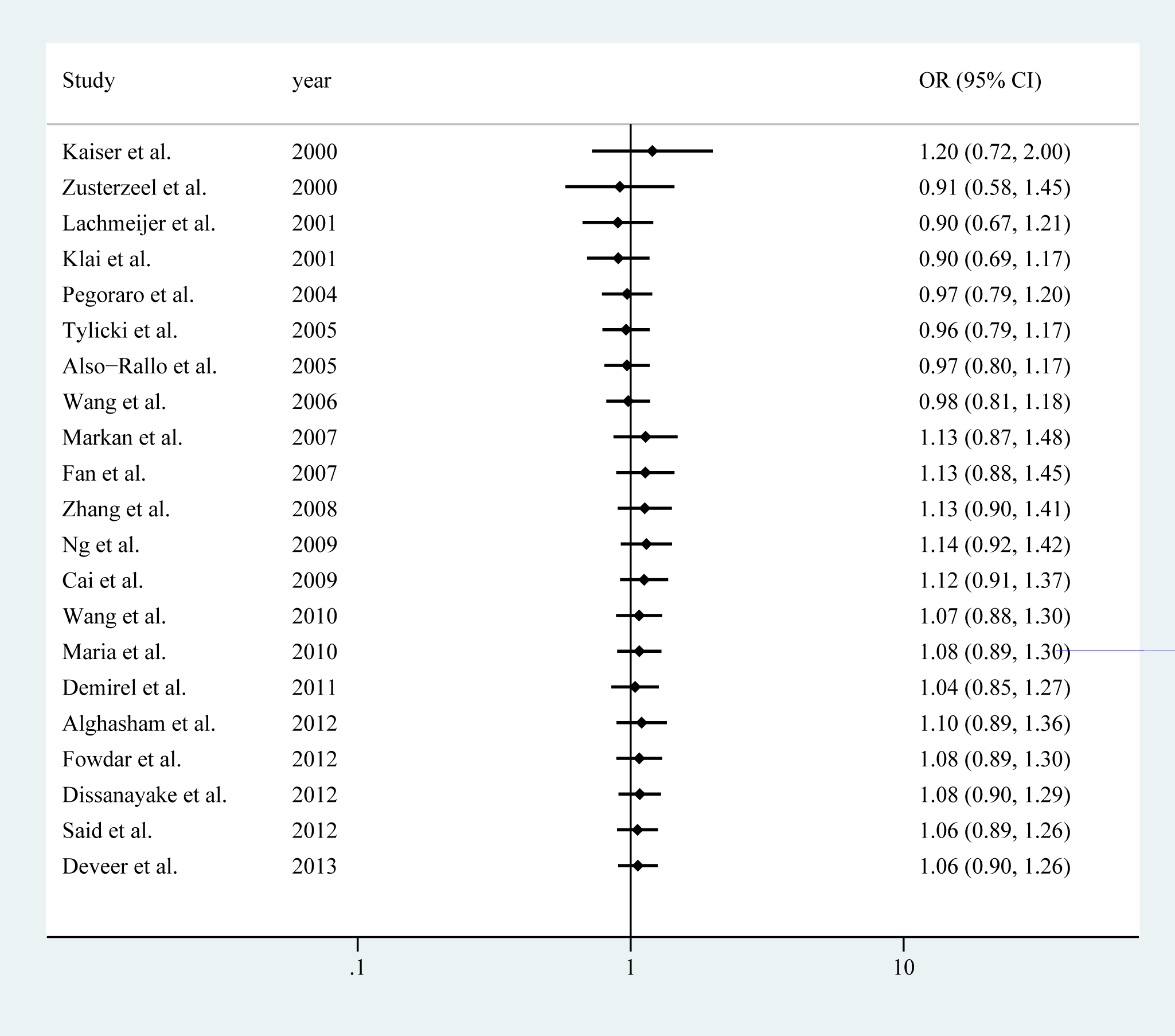

Supplement: Figure S12 — The cumulative forest plot of OR with 95% CI for MTHFR A1298C polymorphism and H & HIP in dominant model. (TIF) [file pone.0087497.s012.tif]
