# Supplementary material for: Associations of MTHFR Gene Polymorphisms with Hypertension and Hypertension in Pregnancy: A Meta-Analysis from 114 Studies with 15411 Cases and 21970 Controls
Source: PLoS One. 2014 Feb 5;9(2):e87497. doi: 10.1371/journal.pone.0087497 (PMC3914818; doi:10.1371/journal.pone.0087497)
Supplement: Table S1 — Baseline characteristics of qualified studies in this meta-analysis. (DOC) [file pone.0087497.s013.doc]

**Table S1.** Baseline characteristics of qualified studies in this meta-analysis.

|  |  |  | **Controls** |  | **Mean age of** | **Gender distributions in** | **Diagnosis** | **Genotyping** |  |
| --- | --- | --- | --- | --- | --- | --- | --- | --- | --- |
| **Author** | **Year** | **Country** | **Source** | **Ethnicity** | **Cases/controls** | **Cases/controls (male%)** | **standard** | **method** | **Matching criteria** |
| **H** |  |  |  |  |  |  |  |  |  |
| Nakata et al. | 1998 | Japan | HB | East Asians | NR/NR | NR/NR | 160/95 | PCR-RFLP | age, sex |
| Zhan et al. | 2000 | China | PB | East Asians | 57.9/50.6 | 52.8/42.4 | 140/90 | PCR-RFLP | area |
| Benes et al. | 2001 | Czech | HB | Caucasians | 55.6/54.9 | 79.8/72.7 | 140/90 | PCR-RFLP | NR |
| Wang et al. | 2002 | China | HB | East Asians | 62.1/60.1 | 14.3/11.1 | 140/90 | PCR-RFLP | Ethnicity, area |
| Rodríguez et al. | 2003 | Spain | PB | Caucasians | 58.6/57.5 | 71.6/75.4 | 140/90 | PCR-RFLP | age, sex, history of disease |
| Heux et al. | 2004 | Australia | HB | Caucasians | 55.0/55.0 | 36.0/36.0 | 140/95 | PCR-RFLP | age, sex, ethnicity |
| Liu et al. | 2004 | China | PB | East Asians | 67.2/66.3 | 50.0/50.0 | 140/90 | PCR-RFLP | ethnicity, area |
| Tylicki et al. | 2005 | Mixed | HB | Caucasians | 59.4/52.1 | 63.3/63.3 | NS | PCR-RFLP | age, sex, ethnicity |
| Lwin et al. | 2006 | Japan | PB | East Asians | NR/NR | NR/NR | 140/90 | PCR-RFLP | NR |
| Li et al. | 2006 | China | HB | East Asians | 60.3/55.5 | 37.8/43.3 | 140/90 | PCR-RFLP | NR |
| Hui et al. | 2007 | Japan | PB | East Asians | 51.1/51.5 | 65.1/67.2 | 160/100 | Taqman | area |
| Markan et al. | 2007 | Indian | HB | Indians and | 47.7/46.2 | 54.3/51.9 | 140/90 | PCR-RFLP | age, sex, ethnicity |
|  |  |  |  | Sri Lankans |  |  |  |  |  |
| Xing et al. | 2007 | China | HB | East Asians | 48.9/48.5 | 56.6/38.5 | 140/90 | PCR-RFLP | ethnicity, area, age |
| Deng et al. | 2007 | China | PB | East Asians | 47.8/46.5 | 38.4/37.7 | 140/90 | PCR-RFLP | ethnicity, area, sex, age |
| Hu et al. | 2007 | China | HB | East Asians | 56.7/55.3 | 47.2/45.2 | 140/90 | MassArray | age, sex, ethnicity, area |
| Lin et al. | 2008 | China | HB | East Asians | 60.6/59.0 | 62.0/56.9 | 140/90 | PCR-RFLP | age, area, ethnicity |
| Ilhan et al. | 2008 | Turkey | HB | Caucasians | 57.2/57.5 | 35.9/74.0 | 140/90 | PCR-RFLP | age |
| Luo et al. | 2008 | China | HB | East Asians | 63.6/62.0 | 54.3/52.8 | 140/90 | PCR-RFLP | NR |
| Ng et al. | 2009 | English | PB | Caucasians | NR/NR | 44.7/42.5 | 140/90 | PCR-RFLP | NR |
| Fakhrzadeh et al. | 2009 | Iranian | PB | Caucasians | 50.0/34.0 | 31.3/34.2 | 160/95 | PCR-RFLP | NR |
| Cai et al. | 2009 | China | HB | East Asians | NR/NR | NR/NR | 140/90 | PCR-RFLP | sex, age, ethnicity, weight |
| Yu et al. | 2010 | China | HB | East Asians | NR/NR | 0/0 | NS | PCR-RFLP | ethnicity, area |
| Wang et al. | 2010 | China | PB | East Asians | 49.5/40.8 | 49.3/45.3 | 140/90 | PCR-RFLP | ethnicity, area |
| Demirel et al. | 2011 | Turkey | PB | Caucasians | 38.3/37.1 | NR/NR | 140/90 | PCR-RFLP | age, sex |
| Mendilcioglu et al. | 2011 | Turkey | HB | Caucasians | 31.4/27.2 | 0/0 | 140/90 | PCR-RFLP | NR |
| Jin et al. | 2011 | China | HB | East Asians | 66.0/65.0 | 52.6/50.5 | 140/90 | PCR-RFLP | ethnicity, area |
| Ma et al. | 2011 | China | HB | East Asians | 62.5/60.8 | 64.8/60.0 | 140/90 | gene chip | ethnicity, area |
| Liu et al. | 2011 | China | HB | East Asians | 48.3/45.1 | 50.7/56.3 | 140/90 | PCR-RFLP | ethnicity, area |
| Su et al. | 2011 | China | HB | East Asians | 28.3/27.4 | 0/0 | 140/90 | PCR-RFLP | ethnicity, area |
| Alghasham et al. | 2012 | Saudi Arabia | PB | Caucasians | 50.9/47.7 | 67.5/56.8 | 140/90 | Taqman | area |
| Fowdar et al. | 2012 | Australia | PB | Caucasians | 63.1/61.0 | 46.7/46.7 | 140/90 | PCR-RFLP | age, sex, area, ethnicity |
| Yin et al. | 2012 | China | PB | East Asians | 48.5/48.5 | 49.3/49.6 | 140/90 | PCR-RFLP | ethnicity, sex, age |
| Zhang et al. | 2012 | China | PB | East Asians | 50.3/49.9 | 73.0/72.7 | 140/90 | PCR-RFLP | ethnicity, area |
| Cao et al. | 2012 | China | HB | East Asians | 78.9/74.5 | 64.3/54.1 | 140/90 | PCR-RFLP | age, sex, ethnicity |
| Fridman et al. | 2013 | Argentina | HB | Latinos | 56.3/41.0 | 0/0 | 140/90 | PCR-RFLP | NR |
| Yao et al. | 2013 | China | HB | East Asians | 63.4/62.8 | 58.0/58.7 | 140/90 | PCR-RFLP | sex, age, area, ethnicity |
| Yang et al. | 2013 | China | HB | East Asians | 47.0/45.2 | 44/46.5 | 140/90 | PCR-RFLP | ethnicity area |
| Bayramoglu et al. | 2013 | Turkey | HB | Caucasians | 57.0/55.5 | 32.8/63.64 | 140/90 | PCR-RFLP | NR |
| **HIP** |  |  |  |  |  |  |  |  |  |
| Sohda et al. | 1997 | Japan | HB | East Asians | 31.0/30.6 | 0/0 | NR | PCR-RFLP | NR |
| Grandone et al. | 1999 | Italy | HB | Caucasians | 30.6/33.8 | 0/0 | 140/90 | PCR-RFLP | ethnicity |
| Chikosi et al. | 1999 | South Africa | HB | Black | 25/24 | 0/0 | 140/90 | PCR-RFLP | NR |
|  |  |  |  | Africans |  |  |  |  |  |
| O' Shaughnessy et al. | 1999 | UK | HB | Caucasians | NR/NR | 0/0 | 140/90 | PCR-RFLP | age, area |
| Powers et al. | 1999 | USA | HB | Caucasians | 27.3/22.6 | 0/0 | 140/90 | PCR-RFLP | ethnicity |
| Kobashi et al. | 2000 | Japan | HB | East Asians | 30.9/29.8 | 0/0 | 140/90 | PCR-RFLP | NR |
| Kaiser et al. | 2000 | Australia | HB | Caucasians | NR/NR | 0/0 | 140/90 | PCR-RFLP | ethnicity |
| Jr et al. | 2000 | Hungary | PB | Caucasians | 27.3/26.5 | 0/0 | 160/100 | PCR-RFLP | age, parity |
| Rajkovic et al. | 2000 | Zimbabwe | HB | Black | NR/NR | 0/0 | 140/90 | PCR-RFLP | NR |
|  |  |  |  | Africans |  |  |  |  |  |
| Zusterzeel et al. | 2000 | Netherlands | PB | Caucasians | 28.0//51.2 | 0/0 | 140/90 | PCR-RFLP | NR |
| Laivuori et al. | 2000 | Finland | HB | Caucasians | 29.6/30.0 | 0/0 | 140/90 | PCR-RFLP | area, ethnicity |
| Murphy et al. | 2000 | Ireland | HB | Caucasians | NR/NR | 0/0 | 140/90 | PCR-RFLP | NR |
| Kupferminc et al. | 2000 | Israel | HB | Caucasians | 26.0/28.5 | 0/0 | 160/110 | PCR-RFLP | age, ethnicity |
| Li et al. | 2000 | China | HB | East Asians | NR/NR | 0/0 | 140/90 | PCR-RFLP | NR |
| Kim et al. | 2001 | Korea | HB | East Asians | NR/NR | 0/0 | 140/90 | PCR-RFLP | ethnicity |
| Livingston et al. | 2001 | USA | HB | Caucasians | 24.5/24.4 | 0/0 | 160/110 | PCR-RFLP | gestational age |
| Lachmeijer et al. | 2001 | Netherlands | PB | Caucasians | NR/NR | 0/0 | 140/90 | PCR-RFLP | area, ethnicity |
| Raijmakers et al. | 2001 | Netherlands | PB | Caucasians | NR/NR | 0/0 | NS/90 | PCR-RFLP | age |
| Wei et al. | 2001 | China | HB | East Asians | 26.8/25.7 | 0/0 | 140/90 | PCR-RFLP | NR |
| Alfirevic et al. | 2001 | UK | HB | Caucasians | 29.0/33.0 | 0/0 | NS | PCR-RFLP | age, parity, gestation |
| Watanabe et al. | 2001 | Japan | HB | East Asians | NR/NR | 0/0 | 140/90 | PCR-RFLP | NR |
| D’Elia et al. | 2002 | Italy | HB | Caucasians | 31.3/32.1 | 0/0 | 140/90 | PCR-RFLP | NR |
| Morrison et al. | 2002 | Scotland | PB | Caucasians | NR/NR | 0/0 | 140/90 | PCR-RFLP | age |
| Prasmusinto et al. (1) | 2002 | German | HB | Caucasians | 29.2/30.0 | 0/0 | 140/90 | PCR-RFLP | age, ethnicity |
| Prasmusinto et al. (2) | 2002 | Croatian | HB | Caucasians | 29.2/30.0 | 0/0 | 140/90 | PCR-RFLP | age, ethnicity |
| Prasmusinto et al. (3) | 2002 | Indonesian | HB | Indians and | 28.8/28.4 | 0/0 | 140/90 | PCR-RFLP | age, ethnicity |
|  |  |  |  | Sri Lankans |  |  |  |  |  |
| Fu et al. | 2003 | China | HB | East Asians | 26.3/25.7 | 0/0 | 140/90 | PCR-RFLP | NR |
| Zhang et al. | 2003 | China | HB | East Asians | 28.5/25.4 | 0/0 | 140/90 | PCR-RFLP | NR |
| Pérez-Mutul et al. | 2004 | Mexico | PB | Latinos | 24.4/27.5 | 0/0 | 140/90 | PCR-RFLP | ethnicity |
| Yilmaz et al. | 2004 | Turkey | HB | Caucasians | 28.0/26.0 | 0/0 | 140/90 | PCR-RFLP | NR |
| Wang et al. | 2004 | China | HB | East Asians | 28.0/28.0 | 0/0 | 140/90 | PCR-RFLP | age, area |
| Williams et al. | 2004 | Peru | HB | Latinos | NR/NR | 0/0 | 140/90 | PCR-RFLP | age |
| Pegoraro et al. | 2004 | South Africa | HB | Black | 26.2/25 | 0/0 | 140/90 | PCR-RFLP | ethnicity |
|  |  |  |  | Africans |  |  |  |  |  |
| Maat et al. | 2004 | Netherlands | HB | Caucasians | 28.0/28.0 | 0/0 | 140/90 | PCR-RFLP | NR |
| Driul et al. | 2005 | Italy | HB | Caucasians | 32.0/32.1 | 0/0 | NR | PCR-RFLP | NR |
| Dávalos et al. | 2005 | Mexico | HB | Latinos | 24.3/26.4 | 0/0 | 140/90 | PCR-RFLP | NR |
| Hernández-Díaz et al. | 2005 | Mixed | PB | Caucasians | NR/NR | 0/0 | NR | PCR-RFLP | age, region |
| Also-Rallo et al. | 2005 | Spain | HB | Caucasians | 30.5/30.0 | 0/0 | 140/90 | PCR-RFLP | area |
| Mello et al. | 2005 | Italy | HB | Caucasians | 33.1/32.4 | 0/0 | 140/90 | PCR-RFLP | area, age |
| Ulukus et al. | 2005 | Turkey | HB | Caucasians | 28.7/28.5 | 0/0 | 140/90 | PCR-RFLP | NR |
| Tian et al. | 2005 | China | HB | East Asians | NR/NR | 0/0 | 140/90 | PCR-RFLP | ethnicity, area, age |
| Jääskeläinen et al. | 2006 | Finland | PB | Caucasians | 28.8/28.7 | 0/0 | 140/90 | PCR-RFLP | area |
| Dalmáz et al. | 2006 | Brazial | HB | Caucasians | 27.8/27.3 | 0/0 | 160/110 | PCR-RFLP | area, age |
| Yalinkaya et al. | 2006 | Turkey | HB | Caucasians | 30.3/29.7 | 0/0 | 140/90 | PCR-RFLP | area |
| Wang et al. | 2006 | China | HB | East Asians | 27.9/27.4 | 0/0 | 140/90 | PCR-RFLP | ethnicity, area |
| Demir et al. | 2006 | Turkey | HB | Caucasians | NR/NR | 0/0 | 140/90 | PCR | NR |
| Nagy et al. | 2007 | Hungary | HB | Caucasians | 29.5/28.3 | 0/0 | 160/90 | Taqman | NR |
| Dusse et al. | 2007 | Brazial | HB | Latinos | NR/NR | 0/0 | 160/NS | PCR-RFLP | age, socio-economic status |
| Stonek et al. | 2007 | Austria | HB | Caucasians | 23.7/23.9 | 0/0 | NS | microarray | NR |
| Zhang et al. | 2007 | China | HB | East Asians | 29.2/28.7 | 0/0 | NS | PCR-RFLP | NR |
| Fan et al. | 2007 | China | HB | East Asians | 26.4/27.4 | 0/0 | 140/90 | PCR-RFLP | ethnicity, area |
| Canto et al. | 2008 | Mexico | HB | Latinos | 22.0/21.9 | 0/0 | 140/90 | Taqman | area |
| Muetze et al. | 2008 | German | HB | Caucasians | 30.0/30.9 | 0/0 | 140/90 | Taqman | area, ethnicity |
| Ding et al. | 2008 | China | HB | East Asians | NR/NR | 0/0 | 140/90 | PCR-RFLP | NR |
| Wang et al. | 2008 | China | HB | East Asians | 30.0/29.0 | 0/0 | 140/90 | PCR-RFLP | ethnicity, area |
| Zhang et al. | 2008 | China | HB | East Asians | 27.5/25.4 | 0/0 | 140/90 | PCR-RFLP | age, gestational age |
| Stiefel et al. | 2009 | Spain | HB | Caucasians | 29.7/28.8 | 0/0 | 140/90 | PCR-RFLP | area, ethnicity |
| Kahn et al. | 2009 | Canada | HB | Caucasians | NR/NR | 0/0 | 90 | PCR-RFLP | area |
| Shen et al. | 2009 | China | HB | East Asians | 27.9/29.7 | 0/0 | 140/90 | PCR-RFLP | NR |
| Rojas et al. | 2010 | Mexico | HB | Latinos | 24.0/22.0 | 0/0 | 140/90 | PCR-RFLP | NR |
| Zhong et al. | 2010 | China | HB | East Asians | 24.2/23.8 | 0/0 | 140/90 | PCR-RFLP | area, age |
| Procopciuc et al. | 2010 | Romanian | HB | Latinos | 27.7/NR | 0/0 | 140/90 | PCR-RFLP | age |
| Aggarwal et al. | 2011 | Indian | HB | Indians and | 25.8/25.0 | 0/0 | 140/90 | PCR-RFLP | age |
|  |  |  |  | Sri Lankans |  |  |  |  |  |
| Saravani et al. | 2011 | Iran | HB | Caucasians | 28.4/26.4 | 0/0 | 140/90 | PCR-RFLP | age |
| Klai et al. | 2011 | Tunisia | HB | Caucasians | 34.4/35.2 | 0/0 | 140/90 | PCR-RFLP | age, ethnicity |
| Dogan et al. | 2011 | Turkey | HB | Caucasians | 28.9/25.1 | 0/0 | 140/90 | Taqman | NR |
| Mislanova et al. | 2011 | Ukraine | HB | Caucasians | 27.6/25.9 | 0/0 | 140/90 | PCR-RFLP | ethnicity |
| Lykke et al. | 2012 | Denmark | PB | Caucasians | 30.2/30.3 | 0/0 | 160/110 | Taqman | NR |
| Dissanayake et al. | 2012 | Sri Lanka | HB | Indians and | 27.0/27.1 | 0/0 | 140/90 | PCR-RFLP | age, ethnicity |
|  |  |  |  | Sri Lankans |  |  |  |  |  |
| Ibrahim et al. | 2012 | Egypt | HB | Caucasians | 24.1/23.7 | 0/0 | 140/90 | PCR-RFLP | age |
| Said et al. | 2012 | Australia | HB | Caucasians | 29.8/30.0 | 0/0 | 160/110 | Taqman | age, ethnicity |
| Coral-Vázquez et al. | 2013 | Mexico | HB | Latinos | 25.1/24.6 | 0/0 | 160/110 | Taqman | area, ethnicity |
| Kaur et al. | 2013 | Indian | HB | Indians and | 24.9/25.3 | 0/0 | 140/90 | PCR-RFLP | age, area, ethnicity |
|  |  |  |  | Sri Lankans |  |  |  |  |  |
| Rahimi et al. | 2013 | Iran | HB | Caucasians | 29.0/27.4 | 0/0 | 140/90 | PCR-RFLP | NR |
| Deveer et al. | 2013 | Turkey | HB | Caucasians | 28.8/27.2 | 0/0 | 140/90 | PCR | NR |
| Alaniz et al | 2013 | Mexico | HB | Latinos | 24.7/24.7 | 0/0 | 140/90 | RT-PCR | age, gestational age |

Abbreviation: H, hypertension; HIP, hypertension in pregnancy; HB, hospital based; PB, populaton based; PCR-RFLP, polymerase chain reaction-restriction fragment length polymorphism; NR, data not reported.
