# Supplementary material for: Associations of MTHFR Gene Polymorphisms with Hypertension and Hypertension in Pregnancy: A Meta-Analysis from 114 Studies with 15411 Cases and 21970 Controls
Source: PLoS One. 2014 Feb 5;9(2):e87497. doi: 10.1371/journal.pone.0087497 (PMC3914818; doi:10.1371/journal.pone.0087497)
Supplement: Table S2 — Distribution of genotype and allele frequencies of the MTHFR C677T polymorphism. (DOC) [file pone.0087497.s014.doc]

**Table S2.** Distribution of genotype and allele frequencies of the *MTHFR* C677T polymorphism.

|  | **Genotype distribution** | | | | | | |  | **Allele frequency** | | | | |  |  |  |  |
| --- | --- | --- | --- | --- | --- | --- | --- | --- | --- | --- | --- | --- | --- | --- | --- | --- | --- |
|  | **Cases, n** | | |  | **Controls, n** | | |  | **Cases, %** | |  | **Controls, %** | |  | **Sample size** | |  |
| **Author** | **CC** | **CT** | **TT** |  | **CC** | **CT** | **TT** |  | **C** | **T** |  | **C** | **T** | ***P*_HWE_** | **case** | **control** | **NOS scores** |
| **H** |  |  |  |  |  |  |  |  |  |  |  |  |  |  |  |  |  |
| Nakata et al. | 63 | 91 | 19 |  | 65 | 83 | 36 |  | 62.7 | 37.3 |  | 57.9 | 42.1 | 0.3099 | 173 | 184 | 5 |
| Zhan et al. | 44 | 68 | 15 |  | 62 | 84 | 24 |  | 61.4 | 38.6 |  | 61.2 | 38.8 | 0.6001 | 127 | 170 | 9 |
| Benes et al. | 73 | 93 | 27 |  | 86 | 106 | 17 |  | 61.9 | 38.1 |  | 66.5 | 33.5 | 0.0454 | 193 | 209 | 4 |
| Wang et al. | 17 | 51 | 37 |  | 14 | 23 | 9 |  | 40.5 | 59.5 |  | 55.4 | 44.6 | 0.9354 | 105 | 46 | 7 |
| Rodríguez et al. | 83 | 115 | 34 |  | 95 | 100 | 20 |  | 60.6 | 39.4 |  | 67.4 | 32.6 | 0.3861 | 232 | 215 | 9 |
| Heux et al. | 87 | 125 | 35 |  | 105 | 119 | 25 |  | 60.5 | 39.5 |  | 66.1 | 33.9 | 0.2988 | 247 | 249 | 7 |
| Liu et al. | 29 | 45 | 26 |  | 31 | 50 | 19 |  | 51.5 | 48.5 |  | 56.0 | 44.0 | 0.8838 | 100 | 100 | 7 |
| Tylicki et al. | 40 | 39 | 11 |  | 42 | 38 | 10 |  | 66.1 | 33.9 |  | 67.8 | 32.2 | 0.7517 | 90 | 90 | 7 |
| Lwin et al. | 39 | 58 | 19 |  | 64 | 117 | 38 |  | 58.6 | 41.4 |  | 55.9 | 44.1 | 0.2151 | 116 | 219 | 9 |
| Li et al. | 25 | 14 | 6 |  | 21 | 7 | 2 |  | 71.1 | 28.9 |  | 81.7 | 18.3 | 0.2266 | 45 | 30 | 4 |
| Hui et al. | 83 | 129 | 49 |  | 104 | 123 | 44 |  | 56.5 | 43.5 |  | 61.1 | 38.9 | 0.4542 | 261 | 271 | 6 |
| Markan et al. | 105 | 40 | 8 |  | 105 | 28 | 0 |  | 81.7 | 18.3 |  | 89.5 | 10.5 | 0.1749 | 153 | 133 | 7 |
| Xing et al. | 202 | 300 | 184 |  | 182 | 222 | 105 |  | 51.3 | 48.7 |  | 57.6 | 42.4 | 0.0155 | 686 | 509 | 6 |
| Deng et al. | 108 | 35 | 8 |  | 91 | 40 | 7 |  | 83.1 | 16.9 |  | 80.4 | 19.6 | 0.3529 | 151 | 138 | 8 |
| Hu et al. | 55 | 39 | 16 |  | 61 | 42 | 12 |  | 67.7 | 32.3 |  | 71.3 | 28.7 | 0.2488 | 110 | 115 | 5 |
| Lin et al. | 19 | 27 | 4 |  | 73 | 44 | 6 |  | 65.0 | 35.0 |  | 77.2 | 22.8 | 0.8479 | 50 | 123 | 7 |
| Ilhan et al. | 36 | 32 | 10 |  | 72 | 26 | 2 |  | 66.7 | 33.3 |  | 85.0 | 15.0 | 0.8446 | 78 | 100 | 8 |
| Luo et al. | 260 | 151 | 31 |  | 138 | 51 | 6 |  | 75.9 | 24.1 |  | 83.8 | 16.2 | 0.6298 | 442 | 195 | 5 |
| Ng et al. | 14 | 14 | 10 |  | 40 | 32 | 8 |  | 55.3 | 44.7 |  | 70.0 | 30.0 | 0.6702 | 38 | 80 | 7 |
| Fakhrzadeh et al. | 99 | 44 | 17 |  | 36 | 31 | 9 |  | 75.6 | 24.4 |  | 67.8 | 32.2 | 0.5628 | 160 | 76 | 7 |
| Cai et al. | 77 | 44 | 9 |  | 31 | 7 | 1 |  | 76.2 | 23.8 |  | 88.5 | 11.5 | 0.4507 | 130 | 39 | 7 |
| Yu et al. | 52 | 44 | 12 |  | 89 | 27 | 9 |  | 68.5 | 31.5 |  | 82.0 | 18.0 | 0.0027 | 108 | 125 | 4 |
| Mendilcioglu et al. | 37^*^ |  | 4 |  | 32^*^ |  | 6 |  |  |  |  |  |  |  | 41 | 38 | 7 |
| Jin et al. | 215 | 140 | 50 |  | 204 | 144 | 52 |  | 70.4 | 29.6 |  | 69.0 | 31.0 | 0.0015 | 405 | 400 | 6 |
| Ma et al. | 6 | 115 | 1 |  | 0 | 44 | 1 |  | 52.0 | 48.0 |  | 48.9 | 51.1 | 0.0000 | 122 | 45 | 6 |
| Liu et al. | 54 | 59 | 33 |  | 61 | 39 | 12 |  | 57.2 | 42.8 |  | 71.9 | 28.1 | 0.1421 | 146 | 112 | 6 |
| Su et al. | 32 | 11 | 11 |  | 331 | 154 | 28 |  | 69.4 | 30.6 |  | 79.5 | 20.5 | 0.0775 | 54 | 513 | 4 |
| Alghasham et al. | 73 |  | 50^#^ |  | 185 |  | 65^#^ |  |  |  |  |  |  |  | 123 | 250 | 6 |
| Fowdar et al. | 170 | 174 | 33 |  | 175 | 183 | 35 |  | 68.2 | 31.8 |  | 67.8 | 32.2 | 0.1863 | 377 | 393 | 7 |
| Yin et al. | 244 | 358 | 68 |  | 322 | 309 | 51 |  | 63.1 | 36.9 |  | 69.9 | 30.1 | 0.047 | 670 | 682 | 9 |
| Zhang et al. | 128 | 53 | 8 |  | 117 | 41 | 7 |  | 81.7 | 18.3 |  | 83.3 | 16.7 | 0.1756 | 189 | 165 | 7 |
| Cao et al. | 33 | 53 | 26 |  | 49 | 68 | 30 |  | 53.1 | 46.9 |  | 56.5 | 43.5 | 0.4736 | 112 | 147 | 5 |
| Fridman et al. | 29 | 40 | 6 |  | 71 | 64 | 15 |  | 65.3 | 34.7 |  | 68.7 | 31.3 | 0.9174 | 75 | 150 | 6 |
| Yao et al. | 32 | 69 | 49 |  | 61 | 67 | 22 |  | 44.3 | 55.7 |  | 63.0 | 37.0 | 0.6078 | 150 | 150 | 7 |
| Yang et al. | 39 | 99 | 62 |  | 61 | 89 | 50 |  | 44.2 | 55.8 |  | 52.7 | 47.3 | 0.1291 | 200 | 200 | 6 |
| Bayramoglu et al. | 65 | 38 | 22 |  | 56 | 38 | 5 |  | 67.2 | 32.8 |  | 76.0 | 24.0 | 0.6543 | 125 | 99 | 7 |
| **HIP** | |  |  |  |  |  |  |  |  |  |  |  |  |  |  |  |  |
| Grandone et al. | 105^*^ |  | 34 |  | 180^*^ |  | 36 |  |  |  |  |  |  |  | 139 | 216 | 7 |
| Sohda et al. | 19 | 32 | 16 |  | 134 | 184 | 40 |  | 52.2 | 47.8 |  | 63.1 | 36.9 | 0.049 | 67 | 358 | 4 |
| Chikosi et al. | 86 | 18 | 1 |  | 97 | 13 | 0 |  | 90.5 | 9.5 |  | 94.1 | 5.9 | 0.5101 | 105 | 110 | 5 |
| O' Shaughnessy et al. | 138 | 114 | 31 |  | 99 | 78 | 23 |  | 68.9 | 31.1 |  | 69.0 | 31.0 | 0.2115 | 283 | 200 | 6 |
| Powers et al. | 35 | 49 | 15 |  | 54 | 46 | 14 |  | 60.1 | 39.9 |  | 67.5 | 32.5 | 0.3949 | 99 | 114 | 6 |
| Kobashi et al. | 37 | 52 | 12 |  | 83 | 99 | 33 |  | 62.4 | 37.6 |  | 61.6 | 38.4 | 0.6985 | 101 | 215 | 6 |
| Kaiser et al. | 65 | 68 | 14 |  | 46 | 49 | 14 |  | 67.3 | 32.7 |  | 64.7 | 35.3 | 0.8664 | 147 | 109 | 5 |
| Jr et al. | 46 | 66 | 8 |  | 42 | 53 | 6 |  | 65.8 | 34.2 |  | 67.8 | 32.2 | 0.0421 | 120 | 101 | 8 |
| Rajkovic et al. | 142 | 28 | 1 |  | 151 | 32 | 0 |  | 91.2 | 8.8 |  | 91.3 | 8.7 | 0.195 | 171 | 183 | 9 |
| Zusterzeel et al. | 72 | 74 | 21 |  | 205 | 162 | 36 |  | 65.3 | 34.7 |  | 71.0 | 29.0 | 0.6232 | 167 | 403 | 5 |
| Laivuori et al. | 64 | 45 | 4 |  | 56 | 40 | 7 |  | 76.5 | 23.5 |  | 73.8 | 26.2 | 0.9684 | 113 | 103 | 6 |
| Murphy et al. | 5 | 3 | 3 |  | 214 | 270 | 56 |  | 59.1 | 40.9 |  | 64.6 | 35.4 | 0.0296 | 11 | 540 | 3 |
| Kupferminc et al. | 48^*^ |  | 15 |  | 114^*^ |  | 12 |  |  |  |  |  |  |  | 63 | 126 | 7 |
| Li et al. | 9 | 30 | 18 |  | 44 | 58 | 18 |  | 42.1 | 57.9 |  | 60.8 | 39.2 | 0.8757 | 57 | 120 | 4 |
| Kim et al. | 131 | 117 | 33 |  | 167 | 152 | 41 |  | 67.4 | 32.6 |  | 67.5 | 32.5 | 0.4748 | 281 | 360 | 6 |
| Livingston et al. | 66 | 34 | 10 |  | 61 | 27 | 7 |  | 75.5 | 24.5 |  | 78.4 | 21.6 | 0.1183 | 110 | 95 | 5 |
| Lachmeijer et al. | 22 | 21 | 4 |  | 58 | 51 | 11 |  | 69.1 | 30.9 |  | 69.6 | 30.4 | 0.9649 | 47 | 120 | 8 |
| Klai et al. | 22 | 20 | 2 |  | 61 | 39 | 0 |  | 72.7 | 27.3 |  | 80.5 | 19.5 | 0.0154 | 44 | 100 | 6 |
| Saravani et al. | 82 | 23 | 1 |  | 78 | 21 | 8 |  | 89.2 | 11.8 |  | 82.7 | 17.3 | 0.0011 | 106 | 107 | 6 |
| Raijmakers et al. | 72 | 74 | 21 |  | 205 | 162 | 36 |  | 65.3 | 34.7 |  | 71.0 | 29.0 | 0.6232 | 167 | 403 | 5 |
| Wei et al. | 23 | 13 | 6 |  | 20 | 11 | 5 |  | 70.2 | 29.8 |  | 70.8 | 29.2 | 0.1181 | 42 | 36 | 5 |
| Alfirevic et al. | 56^*^ |  | 7 |  | 42^*^ |  | 2 |  |  |  |  |  |  |  | 63 | 44 | 7 |
| Watanabe et al. | 40 | 59 | 34 |  | 89 | 103 | 32 |  | 52.3 | 47.7 |  | 62.7 | 37.3 | 0.8028 | 133 | 224 | 4 |
| D’Elia et al. | 52^*^ |  | 6 |  | 65^*^ |  | 9 |  |  |  |  |  |  |  | 58 | 74 | 5 |
| Morrison et al. | 169 | 193 | 42 |  | 81 | 66 | 17 |  | 65.7 | 34.3 |  | 69.5 | 30.5 | 0.5176 | 404 | 164 | 7 |
| Prasmusinto et al. (1) | 7 | 7 | 1 |  | 12 | 15 | 7 |  | 70.0 | 30.0 |  | 57.4 | 42.6 | 0.5671 | 15 | 34 | 6 |
| Prasmusinto et al. (2) | 11 | 12 | 2 |  | 18 | 15 | 5 |  | 68.0 | 32.0 |  | 67.1 | 32.9 | 0.514 | 25 | 38 | 6 |
| Prasmusinto et al. (3) | 34 | 6 | 1 |  | 22 | 5 | 0 |  | 90.2 | 9.8 |  | 90.7 | 9.3 | 0.596 | 41 | 27 | 6 |
| Fu et al. | 24 | 53 | 25 |  | 46 | 40 | 14 |  | 49.5 | 50.5 |  | 66.0 | 34.0 | 0.2769 | 102 | 100 | 5 |
| Zhang et al. | 6 | 45 | 22 |  | 12 | 43 | 19 |  | 39.0 | 61.0 |  | 45.3 | 54.7 | 0.1375 | 73 | 74 | 5 |
| Pérez-Mutul et al. | 33 | 66 | 49 |  | 103 | 239 | 148 |  | 44.6 | 55.4 |  | 45.4 | 54.6 | 0.72 | 148 | 490 | 7 |
| Yilmaz et al. | 29 | 28 | 7 |  | 24 | 17 | 6 |  | 67.2 | 32.8 |  | 69.1 | 30.9 | 0.2966 | 64 | 47 | 4 |
| Wang et al. | 53 | 31 | 15 |  | 25 | 24 | 5 |  | 69.2 | 30.8 |  | 68.5 | 31.5 | 0.8243 | 99 | 54 | 4 |
| Williams et al. | 37 | 61 | 25 |  | 62 | 85 | 30 |  | 54.9 | 45.1 |  | 59.0 | 41.0 | 0.9248 | 123 | 177 | 8 |
| Pegoraro et al. | 298 | 50 | 1 |  | 298 | 38 | 2 |  | 92.6 | 7.4 |  | 93.8 | 6.2 | 0.5163 | 349 | 338 | 5 |
| Maat et al. | 78 | 59 | 20 |  | 63 | 75 | 19 |  | 68.5 | 31.5 |  | 64.0 | 36.0 | 0.6443 | 157 | 157 | 6 |
| Driul et al. | 18^*^ |  | 5 |  | 57^*^ |  | 7 |  |  |  |  |  |  |  | 23 | 64 | 4 |
| Dávalos et al. | 13 | 14 | 6 |  | 24 | 27 | 11 |  | 60.6 | 39.4 |  | 60.5 | 39.5 | 0.4835 | 33 | 62 | 5 |
| Hernández-Díaz et al. | 17 |  | 37^#^ |  | 43 |  | 57^#^ |  |  |  |  |  |  |  | 54 | 100 | 8 |
| Also-Rallo et al. | 11 | 24 | 8 |  | 38 | 57 | 27 |  | 53.5 | 46.5 |  | 54.5 | 45.5 | 0.5223 | 43 | 122 | 6 |
| Mello et al. | 729^*^ |  | 79 |  | 793^*^ |  | 15 |  |  |  |  |  |  |  | 808 | 808 | 6 |
| Ulukus et al. | 8 | 6 | 1 |  | 11 | 13 | 0 |  | 73.3 | 26.7 |  | 72.9 | 27.1 | 0.0688 | 15 | 24 | 5 |
| Tian et al. | 34 | 20 | 7 |  | 40 | 12 | 4 |  | 72.1 | 27.9 |  | 82.1 | 17.9 | 0.0437 | 61 | 56 | 6 |
| Jääskeläinen et al. | 78 | 43 | 12 |  | 64 | 42 | 6 |  | 74.8 | 25.2 |  | 75.9 | 24.1 | 0.7927 | 133 | 112 | 7 |
| Dalmáz et al. | 31 | 27 | 17 |  | 76 | 51 | 18 |  | 59.3 | 40.7 |  | 70.0 | 30.0 | 0.0503 | 75 | 145 | 6 |
| Yalinkaya et al. | 84^*^ |  | 16 |  | 88^*^ |  | 12 |  |  |  |  |  |  |  | 100 | 100 | 5 |
| Wang et al. | 26 | 22 | 6 |  | 89 | 27 | 9 |  | 68.5 | 31.5 |  | 82.0 | 18.0 | 0.0027 | 54 | 125 | 6 |
| Demir et al. | 19 | 29 | 8 |  | 43 | 47 | 12 |  | 59.8 | 40.2 |  | 65.2 | 34.8 | 0.8768 | 56 | 102 | 5 |
| Nagy et al. | 49 | 43 | 9 |  | 32 | 35 | 6 |  | 69.8 | 30.2 |  | 67.8 | 32.2 | 0.4014 | 101 | 73 | 5 |
| Dusse et al. | 16 | 12 | 2 |  | 46 | 31 | 6 |  | 73.3 | 26.7 |  | 74.1 | 25.9 | 0.8054 | 30 | 83 | 6 |
| Stonek et al. | 9 | 14 | 2 |  | 669 | 573 | 155 |  | 64.0 | 36.0 |  | 68.4 | 31.6 | 0.0555 | 25 | 1397 | 7 |
| Zhang et al. | 12 | 21 | 20 |  | 10 | 30 | 9 |  | 42.5 | 57.5 |  | 51.0 | 49.0 | 0.1153 | 53 | 49 | 5 |
| Canto et al. | 36 | 66 | 23 |  | 61 | 131 | 82 |  | 55.2 | 44.8 |  | 46.2 | 53.8 | 0.5278 | 125 | 274 | 6 |
| Muetze et al. | 30 | 34 | 7 |  | 35 | 29 | 15 |  | 66.2 | 33.8 |  | 62.7 | 37.3 | 0.0554 | 71 | 79 | 5 |
| Ding et al. | 20 | 37 | 35 |  | 18 | 55 | 16 |  | 41.8 | 58.2 |  | 51.1 | 48.9 | 0.0256 | 92 | 89 | 4 |
| Wang et al. | 6 | 19 | 17 |  | 13 | 40 | 11 |  | 36.9 | 63.1 |  | 51.6 | 48.4 | 0.0445 | 42 | 64 | 6 |
| Zhang et al. | 22 | 21 | 7 |  | 29 | 8 | 3 |  | 65.0 | 35.0 |  | 82.5 | 17.5 | 0.0519 | 50 | 40 | 6 |
| Stiefel et al. | 157^*^ |  | 27 |  | 343^*^ |  | 57 |  |  |  |  |  |  |  | 184 | 400 | 6 |
| Kahn et al. | 105^*^ |  | 8 |  | 379^*^ |  | 64 |  |  |  |  |  |  |  | 113 | 443 | 8 |
| Shen et al. | 20 | 42 | 18 |  | 30 | 21 | 9 |  | 51.3 | 48.8 |  | 67.5 | 32.5 | 0.1172 | 80 | 60 | 5 |
| Rojas et al. | 8 | 9 | 11 |  | 12 | 19 | 10 |  | 44.6 | 55.4 |  | 52.4 | 47.6 | 0.6496 | 28 | 41 | 6 |
| Zhong et al. | 24 | 32 | 11 |  | 26 | 8 | 6 |  | 59.7 | 40.3 |  | 75.0 | 25.0 | 0.0032 | 67 | 40 | 6 |
| Procopciuc et al. | 9 | 10 | 6 |  | 25 | 6 | 2 |  | 56.0 | 44.0 |  | 84.8 | 15.2 | 0.0925 | 25 | 33 | 6 |
| Aggarwal et al. | 160 | 33 | 7 |  | 134 | 58 | 8 |  | 88.3 | 11.8 |  | 81.5 | 18.5 | 0.588 | 200 | 200 | 7 |
| Dogan et al. | 16 | 15 | 3 |  | 17 | 7 | 1 |  | 69.1 | 30.9 |  | 82.0 | 18.0 | 0.7968 | 34 | 25 | 6 |
| Mislanova et al. | 12 | 11 | 5 |  | 21 | 17 | 2 |  | 62.5 | 37.5 |  | 73.8 | 26.3 | 0.5368 | 28 | 40 | 7 |
| Lykke et al. | 113 | 118 | 31 |  | 906 | 793 | 143 |  | 65.6 | 34.4 |  | 70.7 | 29.3 | 0.0912 | 262 | 1842 | 8 |
| Dissanayake et al. | 136 | 36 | 3 |  | 142 | 27 | 2 |  | 88.0 | 12.0 |  | 90.9 | 9.1 | 0.5809 | 175 | 171 | 7 |
| Ibrahim et al. | 9 | 20 | 15 |  | 16 | 28 | 0 |  | 43.2 | 56.8 |  | 68.2 | 31.8 | 0.002 | 44 | 44 | 6 |
| Said et al. | 57 | 46 | 12 |  | 53 | 49 | 13 |  | 69.6 | 30.4 |  | 67.4 | 32.6 | 0.7433 | 115 | 115 | 6 |
| Coral-Vázquez et al. | 38 | 109 | 83 |  | 71 | 166 | 115 |  | 40.2 | 59.8 |  | 43.8 | 56.3 | 0.4324 | 230 | 352 | 7 |
| Kaur et al. | 122 | 19 | 4 |  | 463 | 109 | 21 |  | 90.7 | 9.3 |  | 87.3 | 12.7 | 0 | 145 | 593 | 6 |
| Rahimi et al. | 110 | 72 | 16 |  | 52 | 45 | 4 |  | 73.7 | 26.3 |  | 73.8 | 26.2 | 0.129 | 198 | 101 | 6 |
| Deveer et al. | 29 | 16 | 5 |  | 26 | 18 | 6 |  | 74.0 | 26.0 |  | 70.0 | 30.0 | 0.3124 | 50 | 50 | 5 |
| Alaniz et al. | 62 | 93 | 39 |  | 54 | 97 | 43 |  | 44.1 | 55.9 |  | 52.8 | 47.2 | 0.9642 | 194 | 194 | 6 |

Abbreviation: *MTHFR*, methylenetetrahydrofolate reductase reductase; HWE, Hardy-Weinberg equilibrium; NOS, Newcastle-Ottawa Scale; H, hypertension; HIP, hypertension in pregnancy.

^*^ Genotype counts for CT+CC

^#^ Genotype counts for TT+CT
