# Supplementary material for: Associations of MTHFR Gene Polymorphisms with Hypertension and Hypertension in Pregnancy: A Meta-Analysis from 114 Studies with 15411 Cases and 21970 Controls
Source: PLoS One. 2014 Feb 5;9(2):e87497. doi: 10.1371/journal.pone.0087497 (PMC3914818; doi:10.1371/journal.pone.0087497)
Supplement: Table S3 — Distribution of genotype and allele frequencies of the MTHFR A1298C polymorphism. (DOC) [file pone.0087497.s015.doc]

**Table S3.** Distribution of genotype and allele frequencies of the *MTHFR* A1298C polymorphism.

|  | **Genotype distribution** | | | | | | |  | **Allele frequency** | | | | |  |  |  |  |
| --- | --- | --- | --- | --- | --- | --- | --- | --- | --- | --- | --- | --- | --- | --- | --- | --- | --- |
|  | **Cases, n** | | |  | **Controls, n** | | |  | **Cases, %** | |  | **Controls, %** | |  | **Sample size** | |  |
| **Author** | **AA** | **AC** | **CC** |  | **AA** | **AC** | **CC** |  | **A** | **C** |  | **A** | **C** | ***P*_HWE_** | **case** | **control** | **NOS scores** |
| **H** |  |  |  |  |  |  |  |  |  |  |  |  |  |  |  |  |  |
| Tylicki et al. | 38 | 43 | 9 |  | 36 | 46 | 9 |  | 66.1 | 33.9 |  | 64.8 | 35.2 | 0.3003 | 90 | 91 | 7 |
| Markan et al. | 99 | 43 | 11 |  | 112 | 17 | 4 |  | 78.8 | 21.2 |  | 90.6 | 9.4 | 0.004 | 153 | 133 | 7 |
| Ng et al. | 37 | 35 | 7 |  | 22 | 14 | 3 |  | 69.0 | 31.0 |  | 74.4 | 25.6 | 0.7143 | 79 | 39 | 7 |
| Cai et al. | 71 | 53 | 6 |  | 20 | 17 | 2 |  | 75.0 | 25.0 |  | 73.1 | 26.9 | 0.5009 | 130 | 39 | 7 |
| Wang et al. | 138 | 57 | 8 |  | 139 | 75 | 11 |  | 82.0 | 18.0 |  | 78.4 | 21.6 | 0.8297 | 203 | 225 | 6 |
| Demirel et al. | 25 | 19 | 6 |  | 14 | 33 | 3 |  | 69.0 | 31.0 |  | 61.0 | 39.0 | 0.0062 | 50 | 50 | 4 |
| Alghasham et al. | 50 |  | 73^*^ |  | 144 |  | 106^*^ |  | 0.0 | 0.0 |  | 0.0 | 0.0 |  | 123 | 250 | 6 |
| Fowdar et al. | 165 | 151 | 52 |  | 162 | 173 | 51 |  | 65.4 | 34.6 |  | 64.4 | 35.6 | 0.6539 | 368 | 386 | 7 |
| **HIP** | | | |  |  |  |  |  |  |  |  |  |  |  |  |  |  |
| Kaiser et al. | 53 | 81 | 13 |  | 44 | 53 | 12 |  | 63.6 | 36.4 |  | 64.7 | 35.3 | 0.5027 | 147 | 109 | 4 |
| Zusterzeel et al. | 91 | 68 | 17 |  | 179 | 186 | 38 |  | 71.0 | 29.0 |  | 67.5 | 32.5 | 0.2981 | 176 | 403 | 5 |
| Lachmeijer et al. | 18 | 22 | 7 |  | 45 | 64 | 11 |  | 61.7 | 38.3 |  | 64.2 | 35.8 | 0.0801 | 47 | 120 | 8 |
| Klai et al. | 40 | 0 | 4 |  | 93 | 7 | 0 |  | 90.9 | 9.1 |  | 96.5 | 3.5 | 0.7168 | 44 | 100 | 6 |
| Pegoraro et al. | 265 | 80 | 4 |  | 263 | 67 | 8 |  | 87.4 | 12.6 |  | 87.7 | 12.3 | 0.1424 | 349 | 338 | 5 |
| Also-Rallo et al. | 24 | 19 | 0 |  | 70 | 41 | 11 |  | 77.9 | 22.1 |  | 74.2 | 25.8 | 0.1754 | 43 | 122 | 6 |
| Wang et al. | 31 | 20 | 3 |  | 60 | 36 | 4 |  | 75.9 | 24.1 |  | 78.0 | 22.0 | 0.6245 | 54 | 100 | 6 |
| Fan et al. | 35 | 24 | 5 |  | 36 | 24 | 2 |  | 73.4 | 26.6 |  | 77.4 | 22.6 | 0.3989 | 64 | 62 | 6 |
| Zhang et al. | 29 | 18 | 3 |  | 24 | 14 | 2 |  | 76.0 | 24.0 |  | 77.5 | 22.5 | 0.9819 | 50 | 40 | 6 |
| Procopciuc et al. | 13 | 10 | 2 |  | 19 | 12 | 2 |  | 72.0 | 28.0 |  | 75.8 | 24.2 | 0.9542 | 25 | 33 | 6 |
| Dissanayake et al. | 71 | 89 | 13 |  | 76 | 83 | 12 |  | 66.8 | 33.2 |  | 68.7 | 31.3 | 0.0919 | 173 | 171 | 7 |
| Said et al. | 55 | 49 | 11 |  | 48 | 54 | 13 |  | 69.1 | 30.9 |  | 65.2 | 34.8 | 0.7074 | 115 | 115 | 6 |
| Deveer et al. | 18 | 25 | 7 |  | 20 | 26 | 4 |  | 61.0 | 39.0 |  | 66.0 | 34.0 | 0.262 | 50 | 50 | 5 |

Abbreviation: *MTHFR*, methylenetetrahydrofolate reductase reductase; HWE, Hardy-Weinberg equilibrium; NOS, Newcastle-Ottawa Scale; H, hypertension; HIP, hypertension in pregnancy.

^*^ Genotype counts for CC+AC
